# Distinctive behavioral and neurochemical profile of social stress in male and female adolescent mice

**DOI:** 10.1186/s13293-026-00928-3

**Published:** 2026-05-27

**Authors:** Ezequiel Monferrer, Rebeca Vidal, Tomás Aledón-Catalá, Michele Malaguarnera, Esther O’Shea, José Miñarro, M. Isabel Colado, Marta Rodríguez-Arias

**Affiliations:** 1 Department of Psychobiology, Faculty of Psychology, Universitat de ValènciaRIAPAD RD24/0003/0004 Instituto de Salud Carlos III, Avda. Blasco Ibáñez 21, 7, Valencia, 46010 Spain; 2 Departament of Pharmacology and Toxicology, Faculty of Medicine, Universidad Complutense de Madrid (UCM), Instituto de Investigación Sanitaria Hospital 12 de Octubre (Imas12), Instituto Universitario de Investigación Neuroquímica (IUIN-UCM), Red de Investigación en Atención Primaria de Adicciones (RIAPAd-ISCIII), Madrid, Spain

**Keywords:** Social defeat, Vicarious social defeat, Mice, Female, Tryptophan, Kynurenine, Anxiety, Depression, Corticosterone

## Abstract

**Background:**

Adolescence is a critical developmental period characterized by increased vulnerability to social stress, closely associated with the emergence of long-term neurobiological alterations. The present study aimed to investigate the short- and long-term effects of social stress during adolescence in mice, using the classical social defeat (SD) model in males and the vicarious social defeat (VSD) model in females, and examining the role of the kynurenine (KYN) pathway in resilience and susceptibility phenotypes.

**Methods:**

A total of 264 OF1 mice were exposed to SD or VSD between PND 26 and 35. Animals were classified as resilient or susceptible based on their performance in the social interaction test (SIT). Behavioral assessments evaluating anxiety- and depression-like behaviors, as well as locomotor activity, were conducted in both the short term (adolescence) and long term (adulthood). In addition, corticosterone levels and tryptophan metabolites were measured in multiple brain regions.

**Results:**

Both SD and VSD increased corticosterone levels in both sexes. Depending on the SIT ratio, stressed mice were classified as resilient or susceptible, with females showing a lower proportion of susceptibility than males. In the short term, all defeated male mice exhibited increased locomotor activity, while anxiety-like behaviors were only observed in resilient male animals. Less time spent in back grooming was only observed in defeated adolescent females (both resilient and susceptible). Long-term effects were more limited, with persistent alterations mainly related to anxiety in resilient female mice. Regarding the KYN pathway, females displayed higher kynurenine levels and increased KYN/tryptophan ratios across several brain regions compared with males. Notably, only resilient females showed reduced KYN levels in the striatum. Furthermore, susceptible male mice exhibited an increased serotonin/tryptophan ratio in the cerebellum, limbic forebrain, and striatum compared to control or resilient males.

**Conclusions:**

Overall, these findings indicate that adolescent social stress induces sex-dependent behavioral and neurochemical responses, with susceptibility/resilience associated with specific sex-dependent alterations in the KYN pathway. These results highlight potential biomarkers underlying adaptive responses to stress and may inform the pathophysiology of stress-related mental disorders.

## Plain Language summary

During adolescence, the brain is still developing, making individuals more vulnerable to stress. Social stress experienced during adolescence, such as bullying or social rejection, undoubtedly increases the risk of mental health problems like anxiety and depression in adulthood. This study explored how social stress during adolescence affects behavior and brain chemistry in male and female mice, and why some individuals cope better than others. Researchers used two models of stress. Male mice experienced direct aggression from an aggressive conspecific (social defeat), while in vicarious social defeat, female mice observed the stressful interactions without being physically attacked. After stress exposure, mice were classified as either “resilient” (able to cope and to maintain social behavior) or “susceptible” (more affected and with social avoidance) based on their social behavior. The results showed that both types of stress increased stress hormone levels in both sexes. In the short term, stressed mice were more active, but only resilient mice showed increased anxiety. However, stressed females showed anhedonia behavior. In adulthood, most behavioral effects faded, although some anxiety remained only in resilient females. Tryptophan is an important molecule involved in mood regulation. We observed that susceptible male mice exhibited less serotonin than tryptophan in specific brain regions compared to resilient or control counterparts, while resilient female mice showed less KYN levels in the hippocampus.

In summary, our results suggest that adolescent stress affects males and females differently, and that resilience may be linked to specific brain chemical changes. Understanding these mechanisms could help improve the prevention and treatment of stress-related mental health disorders.

## Highlights

Both SD and VSD activate the HPA axis in adolescent male and female mice, increasing corticosterone levels.

Depending on social behavior, adolescent stressed mice exhibit two phenotypes: susceptible (social avoidance, depressive-like behaviors) or resilient (maintained social interaction), despite similar corticosterone elevations.

Male stressed mice show increased locomotor activity; however, only resilient mice exhibit heightened anxiety, while anhedonia-related behaviors are evident only in females.

In the kynurenine pathway, female mice display higher KYN and serotonin levels compared to males.

The serotonin/tryptophan ratio emerges as a potential biomarker of susceptibility to social stress in male mice.

## Introduction

Adolescence is a critical period marked by profound physical, emotional, and psychological transformations. This life stage is not only essential for acquiring the knowledge, skills, and abilities necessary for adult roles, but also significantly influences long-term health outcomes [1]. Indeed, exposure to stressful situations or repeated stressors can serve as triggers for multiple psychiatric and psychological conditions, such as anxiety and depression [2], cardiometabolic conditions [3], and substance use disorders [4]. In this context, social stress arises from interpersonal relationships and the broader social environment. It is a response to situations perceived as personally significant and challenging, often due to inadequate coping resources [5].

Currently, well-established models have been developed to study social stress, particularly those based on the resident–intruder paradigm [6]. One of the most widely used models is repeated social defeat (SD) [7]. This paradigm involves introducing an intruder rodent into the territory of an aggressive resident, subjecting the intruder to repeated hostile encounters. SD stress triggers significant physiological and endocrine responses, such as elevated corticosterone levels, alterations in serotonergic and dopaminergic neurotransmission, and an intense neuroinflammatory response [8]. Additionally, SD induces behaviors resembling depression and anxiety [9], and compelling evidence has shown that SD increases drug consumption, enhancing the reinforcing effects of cocaine and ethanol [10, 11]. However, SD involves both physical and emotional stress and is not suitable for studies in female rodents [12]. As a result, novel experimental models have emerged in recent years, such as the social defeat witness model. In vicarious social defeat (VSD), an individual rodent (female) observes the SD of two other males, experiencing emotional but not physical stress [13–15]. This model has been shown to replicate key aspects of SD, such as elevated corticosterone levels and behaviors resembling depression and anxiety [13, 16, 17]. More importantly, recent studies have confirmed that VSD also enhances the reinforcing effects of ethanol and cocaine in female mice [18, 19].

Despite the well-established effects of SD, both clinical and preclinical studies highlight a heterogeneous response to stress. A proportion of individuals can maintain normal functioning even after exposure to high levels of stress. In rodent models, the most used test to classify animals as resilient or susceptible is the social interaction test (SIT) [9]. Susceptible mice display depressive-like behaviors, characterized by reduced social interaction, whereas resilient mice maintain interaction levels similar to those of the control group [20, 21]. Interestingly, while resilient animals exhibit fewer depressive-like behaviors, they show corticosterone levels comparable to those of susceptible mice. Even more intriguingly, mice that are susceptible to depressive-like behaviors demonstrate heightened sensitivity to the rewarding effects of cocaine [22], as well as increased ethanol consumption and motivation for this drug [23]. However, these studies have been conducted in adult stressed animals, with few studies evaluating the resilient phenotype in adolescent mice. The limited studies performed to date, conducted exclusively in male mice, indicate that the response to SD is considerably more complex during adolescence. Unlike adults, only a small proportion of defeated adolescent mice (approximately 20%) are fully susceptible or resilient to specific effects of SD [23–25].

The kynurenine (KYN) pathway represents the primary route of tryptophan (TRP) metabolism, with approximately 95% of this essential amino acid being converted into KYN [26]. Increasing evidence suggests that dysregulation and hyperactivation of the KYN pathway are involved in stress-related disorders [27]. Activation of the KYN pathway is driven by stress exposure and inflammatory mediators, which promote the degradation of TRP through this pathway [28–30]. Stress-induced elevations in brain KYN levels are commonly reflected by an increased KYN/TRP ratio, indicating an accelerated conversion of TRP to KYN [31]. Inflammation and metabolic dysregulation have been proposed as key components in the pathophysiology of major depressive disorder [32]. Reduced plasma levels of tryptophan, serotonin, and kynurenine have been reported in patients with major depressive disorder compared to healthy controls [33]. We have previously reported that defeated mice exhibited higher KYN concentrations, although resilient mice showed smaller increases in the cerebellum than susceptible mice [34].

To date, it remains unclear whether exposure to SD stress during adolescence differentially affects the development of affect-related behaviors in the short-term (adolescence) versus the long-term (adulthood) in mice. Nevertheless, the heightened plasticity of the adolescent brain may facilitate enduring changes that persist into adulthood. The present study aimed to investigate the short- and long-term effects of SD during adolescence in both male and female mice, with a particular focus on resilient responses. Depressive-like behaviors were evaluated using the social interaction test (SIT), the splash test, and the tail suspension test (TST). Motor activity and anxiety-like behaviors were assessed using the open field (OF) test and the elevated plus maze (EPM) test. By comparing adolescent male mice subjected to SD with adolescent female mice exposed to VSD, we were able to isolate the psychological component of social stress common to both paradigms from the physical component present only in SD. Based on this comparison, we hypothesized that vicarious social stress is as potent as direct SD in inducing physiological and behavioral alterations. Comparisons between male and female control groups were included a priori to evaluate the presence of baseline sex-dependent differences independent of the experimental treatment. Male control mice were subjected to the same handling and experimental procedures as female controls. Furthermore, all control animals were housed under identical environmental and housing conditions throughout the entire experimental period. Finally, we examined the effects of SD and VSD on the KYN pathway 24 h after the last social encounter, as stress-induced alterations in this pathway have not previously been evaluated in stressed adolescent animals.

## Methods

### Animals

Two hundred sixty-four OF1 mice (Charles River, France), consisting of 101 females and 102 males, were received in our laboratory at postnatal day 21 (PND) in four different batches. Experimental subjects were accommodated in plastic enclosures (27 × 27 × 14 cm) in groups of four. Thirty-one adult (PND 48) OF1 resident mice (Charles River, France) were housed in isolation for at least one month in plastic cages (21 × 32 × 20 cm) to potentiate aggressiveness [35]. Thirty additional OF1 male mice (postnatal day 21, obtained from Charles River, France) were acquired to serve as intruders that underwent SD by the resident mouse. These animals were housed in groups of four in the same conditions as the experimental subjects. Temperature, humidity, and a standard light cycle (white light exposure from 19:00 to 7:00) were regulated within the laboratory. Food and water were available ad libitum to all the mice used in this study, except during behavioral tests. All procedures adhered to the guidelines outlined in the European Council Directive 2010/63/UE regulating animal research and were approved by the local ethics committees of the University of Valencia (number 2022 VSC PEA 0292 type 2 on 20th January 2023).

### Experimental design

**Fig. 1:**
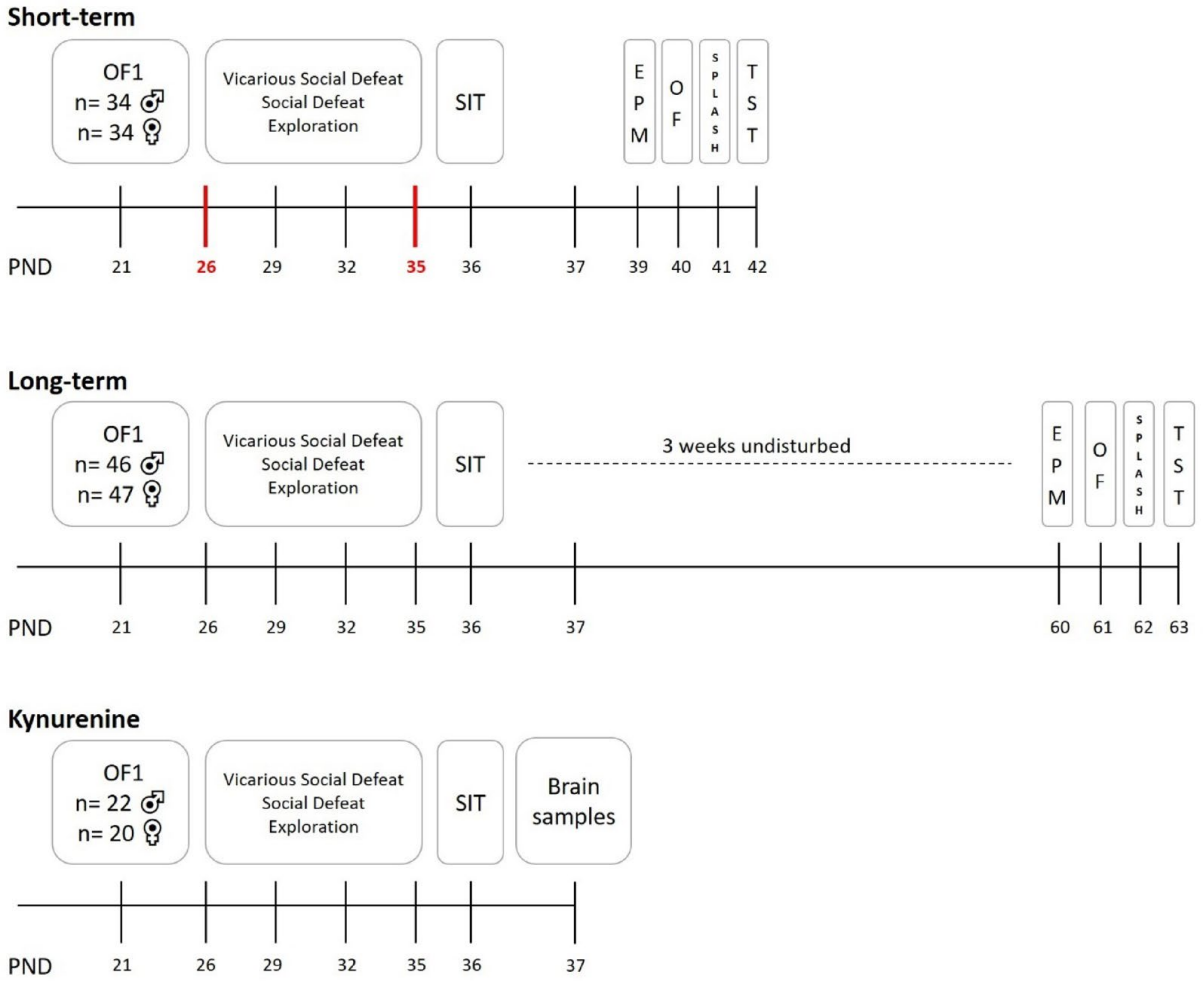
Experimental design. Days highlighted in red indicate when blood samples were collected for corticosterone quantification

In the experimental setup, all mice were received at our laboratory on postnatal day 21 (PND 21) and were housed under standard conditions throughout the duration of the study. For this study, three different sets of mice were employed (Fig. 1): Short-term, Long-term, and Kynurenine. Within each set, mice were exposed to three different conditions from PND 26 to 35. Males were exposed to social defeat (SD) and females to vicarious social defeat (VSD), while control mice of each sex formed the exploration group. Blood samples were collected for corticosterone determination from a subset of short-term animals (*n* = 40), including 10 control and 10 social defeated mice from both sexes. All mice performed the SIT (Social Interaction Test) 24 h after the last SD/VSD episode. From this point onward, the SD/VSD groups were categorized into susceptible and resilient groups, resulting in six experimental groups: male control (*n* = 36); male social defeat resilient (*n* = 41); male social defeat susceptible (*n* = 25); female control (*n* = 40); female social defeat resilient (*n* = 43); female social defeat susceptible (*n* = 18). The short-term set performed several behavioral tests (EPM, OF, Splash and TST) 72 h after the SIT and the long-term set remained undisturbed for 3 weeks until the beginning of the behavioral tests on PND 60. The Kynurenine set was sacrificed on PND 37, the day after the SIT, to analyze TRP metabolism.

### Social defeat in male mice

During adolescence, male mice allocated to the stress/defeat groups underwent a series of four Social Defeat episodes at postnatal day 26, 29, 32 and 35, each one lasting 25 min. The SD paradigm comprised three distinct phases. Initially, the intruder mouse, serving as the experimental subject, was introduced into the home cage of the resident mouse, representing the aggressive opponent, for a 10-minute period [36]. Throughout this initial phase, the intruder was shielded from direct physical harm, yet the presence of wire mesh walls enabled social interactions and typical displays of aggression from the male resident, thereby fostering instigation and provocation [37]. In the second phase, the wire mesh barricade was removed to facilitate direct confrontation between the two mice for a 5-minute interval, during which the interaction was recorded via video and analyzed ethologically. Finally, the wire mesh was reinstated to separate the two mice again for an additional 10 min, allowing for continued social intimidation by the resident. On the other hand, the non-stressed exploration groups underwent an identical protocol in a clean cage without the presence of a resident. For each SD episode, the intruder was exposed to a different resident mouse. The criterion used to define an animal as defeated was the adoption of a specific posture signifying defeat, characterized by an upright submissive position, limp forepaws, upwardly angled head, and retracted ears [35, 38].

### Vicarious social defeat in female mice

The Vicarious Social Defeat (VSD) paradigm was executed following the established methodology as described previously [17]. In the VSD protocol adolescent female mice were exposed to non-physical sensory stimuli (visual, olfactory, and chemosensory) associated with indirectly experiencing the defeat of male OF1 counterparts. During each 15-minute session of VSD, an intruder male mouse was placed into the container of the resident while the VSD female was separated by a mesh wire to only allow vicarious experience (i.e., visual, olfactory, auditory) of the aggressive encounter. Each female experienced four VSD sessions and remained housed with the resident mouse for the next 24 h, separated by a perforated methacrylate barrier (31 × 18 × 0.6 cm) designed to protect from a physical encounter with the male but not from visual, olfactory, and auditory threats. 24 h after the VSD encounter, the female returned to her cage until the next encounter. For each VSD, the female was paired with a different resident mouse. The female control group was exposed to 15 min exploration in a clean cage and then returned to their respective household.

### Social interaction test

The Social Interaction Test (SIT) employed in this study builds upon the method initially described by Berton and colleagues [39], with temporal modifications as outlined by Henriques-Alves and Queiroz [40]. During the dark period and 24 h after the SD or VSD procedure, mice were placed in a different environment (a quiet, dimly illuminated room) from the confrontation and acclimatized for 1 h. Subsequently, each mouse was individually placed in the center of a square arena constructed of black Plexiglas (30 cm on each side, 35 cm high), with behavioral activity recorded via video surveillance (EthoVision XT 11, 50 frames per second; camera positioned above the arena). Mice were allowed to explore the arena twice, for 600 s in each session, during two different experimental sessions. The first session (object session) featured an empty perforated Plexiglas cage (10 × 6.5 × 35 cm) positioned in the middle of one of the arena walls. In the second session (social session), an unfamiliar OF1 male mouse was introduced into the cage as a social stimulus. The arena was cleaned after each session to minimize the olfactory clues. After the first session, the experimental mouse was returned to its cage for 2 min while the unfamiliar mouse was placed in the perforated cage. Arena occupancy during both object and social sessions was quantified and analyzed based on the animals’ horizontal positions using commercial video tracking software (EthoVision XT 11, Noldus). The social preference-avoidance was calculated as a ratio by comparing the time spent in the interaction zone (a 6.5 cm wide corridor surrounding the restraining cage) with and without a social target. A ratio below 1 indicated social avoidance and the mouse was classified as susceptible; otherwise, it indicated social engagement and the mouse was classified as resilient.

### Elevated plus maze

The Elevated Plus Maze (EPM) utilized in this study comprised two open arms (30 × 5 × 0.25 cm) and two enclosed arms (30 × 5 × 15 cm), with the junction of these arms forming a central platform (5 × 5 cm). The floor of the maze was constructed from black Plexiglas, while the walls of the enclosed arms were made of clear Plexiglas. A small edge (0.25 cm) was present on the open arms to enhance grip for the animals. The entire apparatus was elevated to a height of 45 cm above floor level. The experiment was performed in a dimly lit room; mice were transported there one hour before testing. In each trial, subjects were placed on the central platform, facing an open arm, and allowed to explore the maze for a duration of 5 min. Following each trial, the maze was meticulously cleaned with a damp cloth. Behavioral data recorded during the testing period were automatically captured using an automated tracking control software (EthoVision 3.1; Noldus Information Technology, Leesburg, VA). Parameters measured included the frequency of entries, time spent, and percentage of time allocated to each section of the apparatus (open arms, closed arms, central platform). An arm was considered visited when the animal placed all four paws upon it. The number of entries into the open arms, time spent in the open arms, and percentage of open arm entries are commonly employed metrics to characterize anxious behavior.

### Open field

The spontaneous locomotor activity of mice was assessed in an open field environment over a duration of 30 min. The open field test was conducted within a black Plexiglas enclosure measuring 30 × 30 × 15 cm and open at the top. Each animal was introduced into the enclosure, and its activity was automatically monitored and recorded using EthoVision 3.1 software developed by Noldus Information Technology (Leesburg, VA). The parameters analyzed included total distance covered (measured in centimeters), velocity (measured in centimeters per second) and time spent in the center area (measured in seconds).

### Splash test

The Splash test involved the application of a 10% sucrose solution onto the dorsal coat of the mice, which were habituated to the room for 1 h before testing, adhering to the methodology outlined by Hodes and colleagues [41]. Subsequently, the duration of grooming behavior over a 5-minute interval was meticulously observed and assessed by trained evaluators blinded to the experimental group of the animals. The temporal distribution of grooming activity, specifically focusing on leg- and back-grooming, was subjected to detailed analysis. A reduction in back-grooming behavior is indicative of apathetic tendencies, serving as a significant behavioral marker for evaluating the anxious-depressive phenotype in rodent models.

### Tail suspension test

During the Tail Suspension Test (TST), each mouse was individually suspended by adhesive tape affixed 1 cm from the tail tip, positioning them 50 cm above a benchtop surface and away from the vision of other experimental mice, for a duration of 6 min. The behavior displayed by the mice was recorded via video, and subsequently, an objective observer blinded to the experimental group of the animals utilized computerized methods to quantify the behavioral parameter of immobility, which is interpreted as indicative of despair.

### Determination of plasma corticosterone

Blood sampling for corticosterone determination was collected on PND 26 and 35 from the saphenous vein. During this procedure, the animal’s movement was restricted in a tube, and the saphenous vein of the paw was punctured with a needle. Subsequently, the paw was gently massaged until 50 µL of blood was collected into an ice-cold Microvette CB 300 capillary tube (Sarstedt, Nümbrecht, Germany). The blood samples were kept chilled, and plasma was separated from whole blood through centrifugation (5 min, 5000×g) and transferred to sterile 2 mL microcentrifuge tubes. These plasma samples were stored at -80 °C until corticosterone determination.

On the day of the assay, the samples were diluted at a ratio of approximately 1:40 using the Steroid Displacement Reagent mix provided with the kit. Corticosterone levels in the diluted plasma were subsequently analyzed using a corticosterone ELISA kit (Enzo Life Sciences, Farmingdale, NY, USA, Catalog No. ADI-900-097, 96-Well kit), following the manufacturer’s instructions. This analysis was performed using an iMark microplate reader (Bio-Rad, Hercules, CA, USA) and Microplate Manager 6.2 software. Optical density was measured at 405 nm, with a correction at 590 nm. The test’s sensitivity is 0.2 pg/ml.

### Measurement of kynurenine, tryptophan and serotonin

Brain regions (hippocampus, cerebellum, limbic forebrain, and striatum) were dissected and homogenized in deionized water at a 1:5 (w/v) ratio using sonication (Labsonic 2000U, B. Braun Melsungen AG, Germany) at 30% amplitude for 15 s. Homogenates were deproteinized by the addition of 25 µL of 6% perchloric acid per 100 µL of sample. The acidified homogenates were vortexed and incubated at room temperature for 10 min, followed by centrifugation at 16,000×g for 15 min at 4 °C. The resulting supernatants were filtered through 0.2 μm filters (Minisart^®^ RC 4, Sartorius, Thermo Fisher Scientific, MA, USA) for further analysis.

For the quantification of TRP and 5-HT, 20 µL of the filtered supernatant was injected into a reversed-phase high-performance liquid chromatography (HPLC) column (Spherisorb ODS2; 5 μm; 150 × 4.6 mm; Thermo Scientific, Massachusetts, USA). An isocratic elution was performed using a mobile phase consisting of 0.1 M ammonium acetate and 8% methanol, adjusted to pH 3.8 with glacial acetic acid, at a flow rate of 1 mL/min. TRP and 5-HT were detected fluorometrically at excitation/emission wavelengths of 270/360 nm and 290/398 nm, respectively, using a Waters 2475 Multi-Fluorescence Detector (Waters, Milford, MA, USA).

For the quantification of KYN, 60 µL of the supernatant was injected into the same column. KYN was separated using a mobile phase containing 0.1 M sodium acetate and 4% acetonitrile, adjusted to pH 4.6, at a flow rate of 1 mL/min, and detected by UV absorption at 365 nm with a Waters 2487 detector. The retention times for TRP, 5-HT, and KYN were approximately 7, 4.7, and 5 min, respectively. The ratios of KYN and 5-HT to TRP concentrations were calculated and used as indicators of TRP degradation.

### Statistical analysis

Mice were initially classified into resilient and susceptible groups based on their performance in the Social Interaction Test (SIT). This classification involved calculating a ratio: the time spent by an experimental mouse in the interaction zone when a social target was present, divided by the time spent in the interaction zone when the target was absent. A ratio of 1 indicated equal time spent in the presence and absence of a social target. According to the typical behavior of control OF1 mice, animals with a ratio below 1 were classified as susceptible, while those with a ratio of 1 or greater were classified as resilient [42]. To analyze the distribution of SIT results, corticosterone levels, and kynurenine pathway data, a one-way ANOVA was conducted with Stress (Control, Resilient, and Susceptible) as the factor. For the behavioral tests (EPM, OF, Splash, and TST), the same one-way ANOVA was conducted with Stress, separately for the short-term and long-term datasets. Additionally, a two-way ANOVA including Stress and Time (short-term vs. long-term) was performed in each sex to assess behavioral changes over time. Finally, to evaluate sex differences in all measured behaviors and variables, a one-way ANOVA with Sex (male and female) as the factor was performed only for the control groups, as male and female defeated animals were exposed to different experimental protocols. Bonferroni posthoc tests were calculated whenever required. All statistical analyses were performed using SPSS Statistics v.28 and graph design with GraphPad Prism (v8; GraphPad Software Inc., CA, USA). Data were expressed as mean ± SEM and a value of *p* < 0.05 was considered statistically significant.

## Results

### Social interaction test

**Fig. 2:**
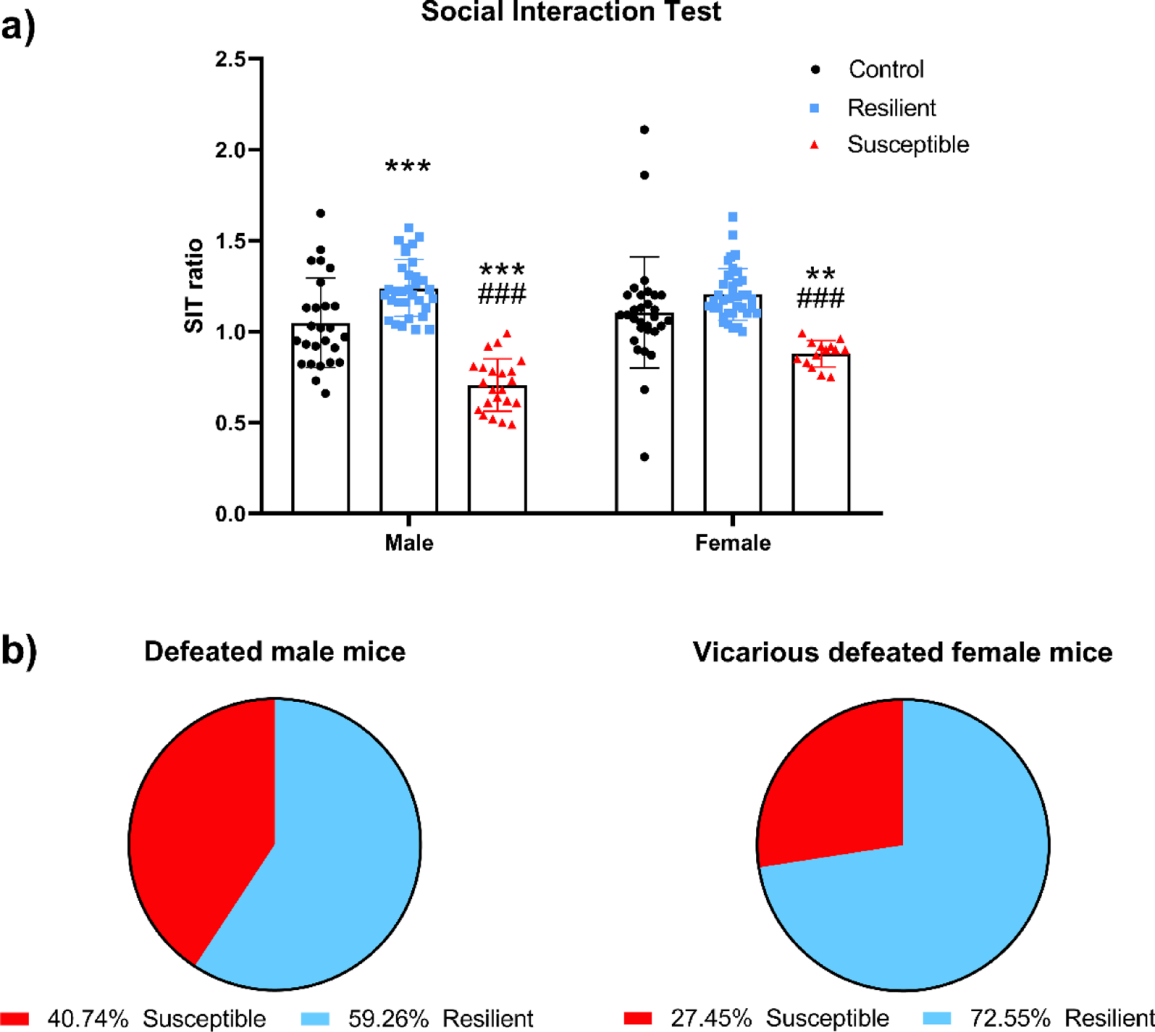
Social defeat stress induces avoidance behavior in susceptible mice. (a) Repeated social defeat stress results in a spectrum of avoidance behavior, divided between susceptible and resilient phenotypes as a function of their social interaction ratio score. (b) Percentages of resilient and susceptible mice in both sexes. Error bars represent means ± SEM. ** *p* < 0.01, *** *p* < 0.001 vs. control mice; ### *p* < 0.001 vs. resilient mice

All mice performed the SIT on PND 36, 24 h after the last SD/VSD. Within the defeated males, 40.7% presented a social interaction ratio < 1 and were classified as susceptible. The remaining 59.3% showed a social interaction ratio ≥ 1 and were considered resilient. Among the stressed females, only 27.5% were classified as susceptible, while 72.5% showed a social interaction ratio ≥ 1 and were classified as resilient (Fig. 2b).

The ANOVA showed an effect of Stress on both males [F [2, 77] = 52.634; *p* < 0.001] and females [F [2, 78] = 11.622; *p* < 0.001]. Post hoc analyses showed that, in both males and females, Resilient and non-stressed (control) animals exhibited higher SIT ratios than Susceptible mice (*p* < 0.001 in all cases, except for non-stressed versus susceptible females, *p* < 0.01). In addition, resilient male mice exhibited higher SIT ratios than non-stressed males (*p* < 0.001) (Fig. 2a).

### Corticosterone Levels

**Fig. 3:**
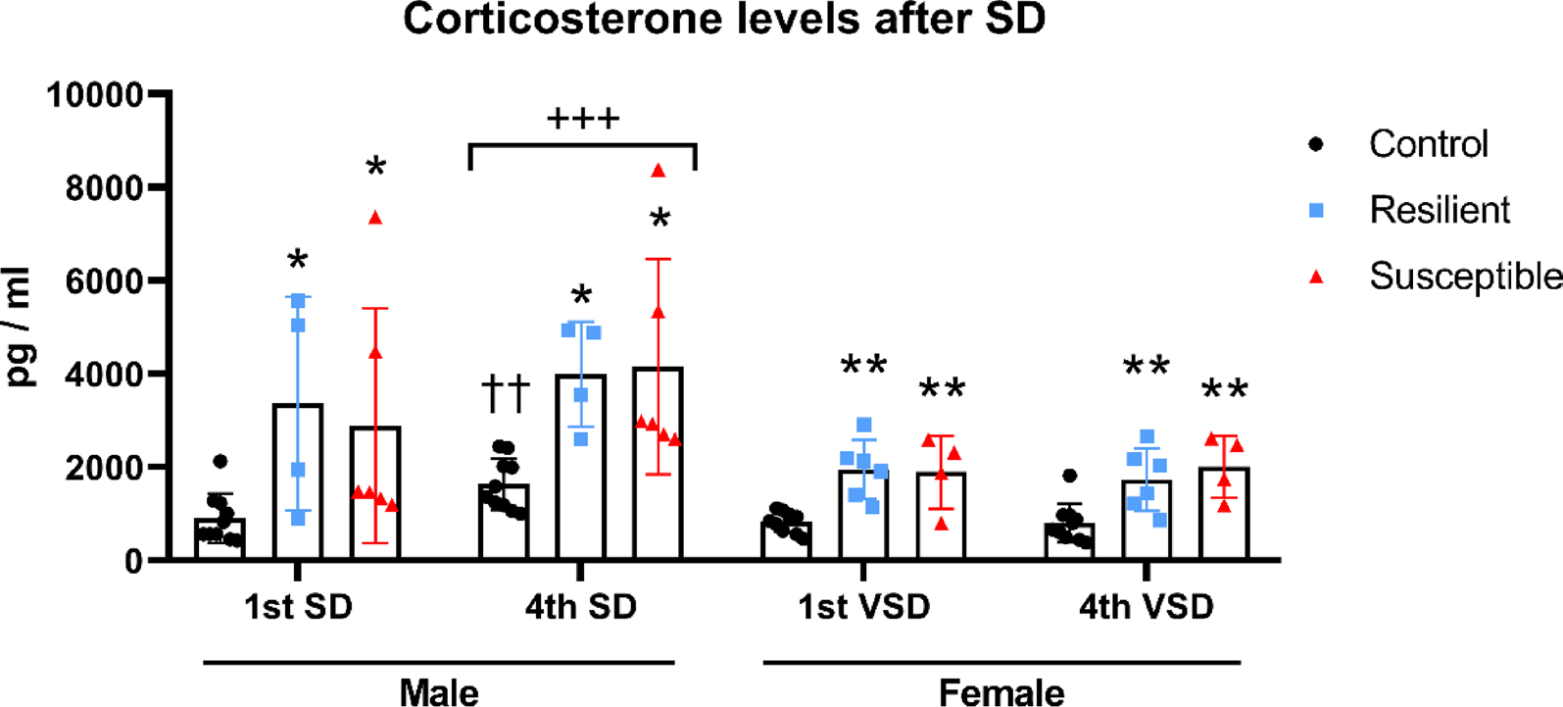
SD and VSD increased corticosterone plasma levels after the 1st and 4th social defeat sessions. The bars represent the mean corticosterone levels and the vertical lines show ± SEM. * *p* < 0.05, ** *p* < 0.01 vs. non-stressed control groups; +++ *p* < 0.001 vs. male groups during the 1st SD; †† *p* < 0.01 vs. the corresponding control female group

The ANOVA for corticosterone levels during the 1st and 4th SD/VSD sessions showed a significant effect of Stress in both males [F [2, 17] = 5.780; *p* = 0.012] and females [F [2, 17] = 12.634; *p* < 0.001]. Resilient and susceptible mice showed higher corticosterone levels than control mice (*p* < 0.05 for males and *p* < 0.01 for females) (Fig. 3). The ANOVA showed an effect of Day [F [1, 17] = 25.530; *p* < 0.001] only in male mice. Male mice showed higher levels during the 4th SD compared with the 1st SD (*p* < 0.001) (Fig. 3). In addition, the ANOVA revealed an interaction effect of Day × Sex [F [1, 18] = 10.486; *p* = 0.005] in control mice. During the 4th SD, control male mice showed higher corticosterone levels than control female mice (*p* < 0.01) (Fig. 3).

### Open field

- Short-term.

**Fig. 4:**
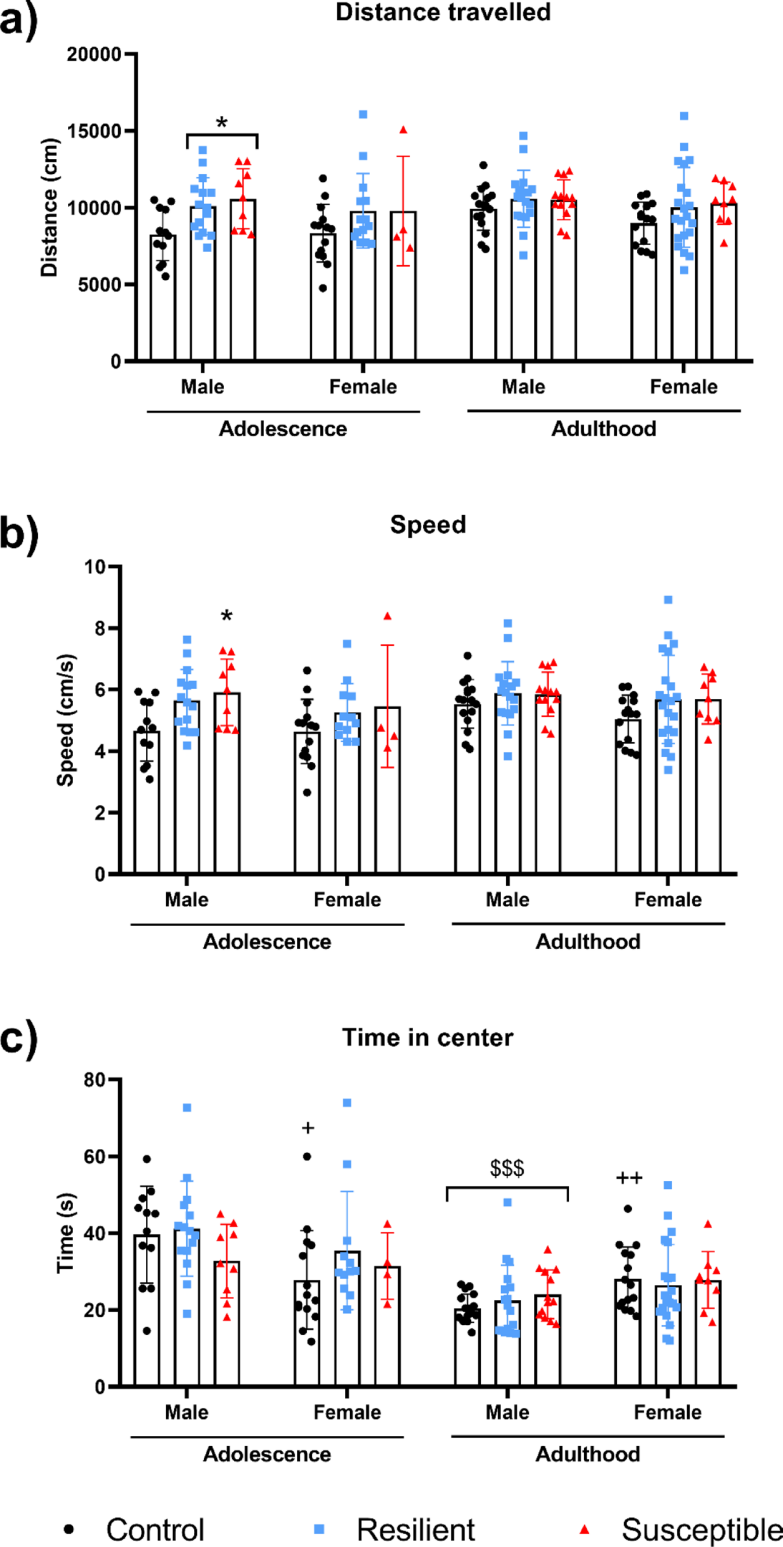
Effects of SD and VSD on the OF test in male and female mice. (**a**) distance traveled; (**b**) speed; and (**c**) time in center. * *p* < 0.05 vs. their respective control group; + *p* < 0.05, ++ *p* < 0.01 vs. their corresponding male groups; $$$ *p* < 0.001 vs. adolescent male groups

In male mice, the ANOVA showed an effect of Stress on distance travelled [F [2, 33] = 5.110; *p* = 0.012] (Fig. 4a) and speed [F [2, 33] = 4.752; *p* = 0.015] (Fig. 4b). All defeated adolescent male mice travelled longer distances than non-stressed mice (*p* < 0.05 in all cases), whereas only susceptible males moved significantly faster than non-stressed mice (*p* < 0.05).

In control mice, the ANOVA showed an effect of Sex on time spent in the center [F [1, 24] = 5.546; *p* = 0.027] (Fig. 4c). Control adolescent male mice spent more time in the center than females (*p* < 0.05).

b) Long-term.

No differences were observed in the OF in adult male or female mice. However, the ANOVA showed an effect of Sex [F [1, 29] = 11.389; *p* = 0.002] in control mice for time spent in the center. Control adult female mice spent more time in the center than control adult males (*p* < 0.01) (Fig. 4c).

For time spent in the center, the ANOVA also showed an effect of Time [F [1, 76] = 53.649; *p* < 0.001] in male mice, which spent a lower percentage of time in the center as adults (*p* < 0.001) (Fig. 4c).

### Elevated plus maze

- Short-term.

In males, the ANOVA showed an effect of Stress on time [F [2, 28] = 8.632; *p* = 0.001] and percentage of time [F [2, 28] = 8.483; *p* = 0.001] spent in the open arms, percentage of open entries [F [2, 28] = 4.493; *p* = 0.020], and number of total entries [F [2, 28] = 3.745; *p* = 0.036]. Resilient male mice spent less time (*p* < 0.001) and a lower percentage of time (*p* < 0.001) in the open arms and made a lower percentage of open entries (*p* < 0.05), but more total entries (*p* < 0.05), than the control non-stressed group (Table 1a).

In control mice, the ANOVA showed an effect of Sex on the number of open entries [F [1, 32] = 6.022; *p* = 0.020] and total entries [F [1, 32] = 8.029; *p* = 0.008]. Control adolescent female mice made a greater number of open (*p* < 0.05) and total entries than control male mice (*p* < 0.01) (Table 1a).

b) Long-term.

In female mice, the ANOVA showed an effect of Stress on time [F [2, 35] = 5.246; *p* = 0.010] and percentage of time [F [2, 35] = 3.551; *p* = 0.039] spent in the open arms. Adult resilient female mice spent less time (*p* < 0.01) and a lower percentage of time (*p* < 0.05) in the open arms than their control group (Table 1b).

In control animals, the ANOVA showed a significant effect of Sex on the number of total entries [F [1, 23] = 6.277; *p* = 0.020]. Adult males made a higher number of total entries than adult control females (*p* < 0.05) (Table 1b).

In female mice, the ANOVA showed an effect of Time on percentage of time spent in the open arms [F [1, 73] = 7.102; *p* = 0.009], time spent in closed arms [F [1, 73] = 13.714; *p* < 0.001], and number of total entries [F [1, 73] = 5.273; *p* = 0.025]. During adolescence, female mice spent a higher percentage of time in the open arms (*p* < 0.01), less time in closed arms (*p* < 0.001), and made a greater number of total entries (*p* < 0.05) compared with adulthood (Table 1).

The ANOVA also showed an interaction effect of Stress × Time on the number of total entries [F [2, 68] = 4.689; *p* = 0.012] in male mice. Control and susceptible male mice made a higher number of total entries as adults (*p* < 0.001 and *p* < 0.05, respectively) (Table 1).

**Table 1:**
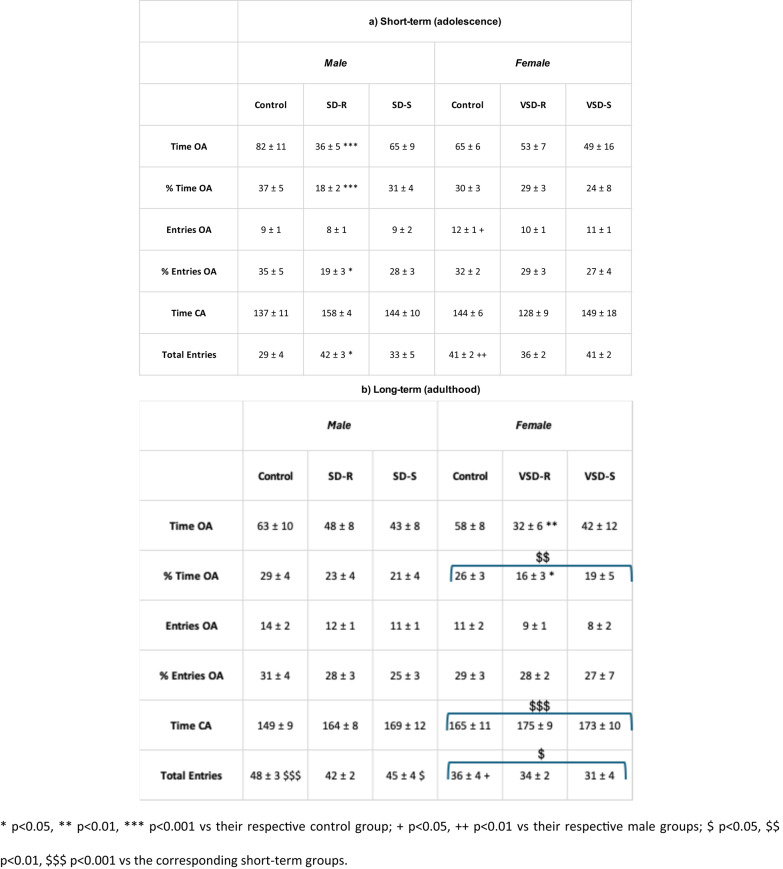
Effects of SD and VSD on the elevated plus maze in (**a**) adolescent and (**b**) adult male and female mice

### Splash test

- Short-term.

For the percentage of time spent in back grooming, the ANOVA showed an effect of Stress in both males [F [2, 33] = 4.051; *p* = 0.027] and females [F [2, 21] = 10.676; *p* < 0.001]. Resilient male mice showed a higher percentage of back grooming than the corresponding control mice (*p* < 0.05), whereas both resilient and susceptible female mice showed a lower percentage of back grooming than the corresponding control females (*p* < 0.01).

**Fig. 5:**
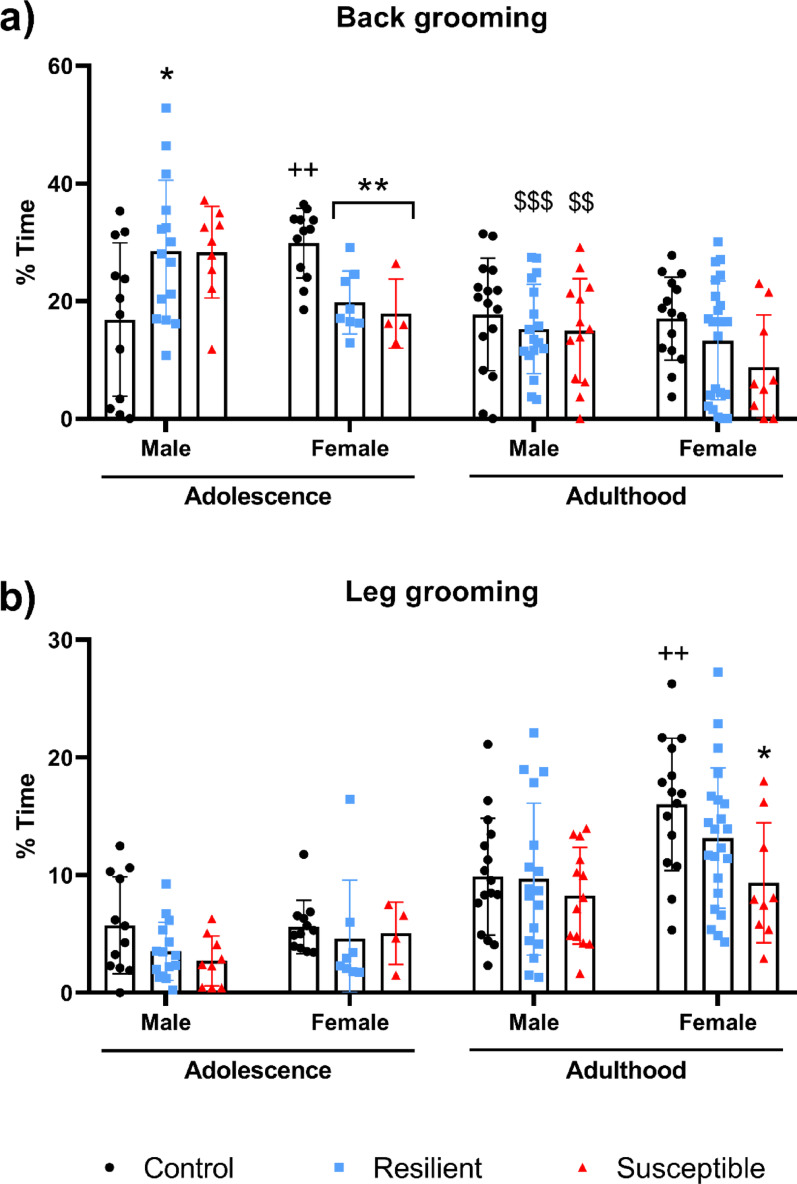
Percentage of time in back (**a**) and leg grooming (**b**) in the Splash test. * *p* < 0.05, ** *p* < 0.01 vs. their corresponding control group; ++ *p* < 0.01 vs. their corresponding male groups; $$ *p* < 0.01, $$$ *p* < 0.001 vs. their corresponding adolescent groups

Additionally, in control animals, the ANOVA showed an effect of Sex [F [1, 22] = 9.922; *p* = 0.005]. Non-stressed control females showed a higher percentage of time spent in back grooming compared with non-stressed male mice (*p* < 0.01) (Fig. 5a).

b) Long-term.

Although no changes were observed in back grooming behavior within adult groups, for the percentage of time spent in leg grooming, the ANOVA showed an effect of Stress [F [2, 43] = 3.869; *p* = 0.028] in female mice. Susceptible female mice engaged in less leg grooming than controls (*p* < 0.05).

In control mice, the ANOVA revealed an effect of Sex [F [1, 29] = 10.445; *p* = 0.003], as control adult female mice spent a higher percentage of time in leg grooming compared with control adult male mice (*p* < 0.01) (Fig. 5b).

To assess changes in behavior depending on age, the ANOVA showed an interaction effect of Stress × Time on the percentage of time spent in back grooming [F [2, 76] = 4.528; *p* = 0.014]. Post hoc analyses revealed that defeated male mice (both resilient and susceptible) spent a higher percentage of time in back grooming during adolescence than in adulthood (*p* < 0.001 for resilient and *p* < 0.01 for susceptible) (Fig. 5a).

### Tail suspension test

- Short-term.

**Fig. 6:**
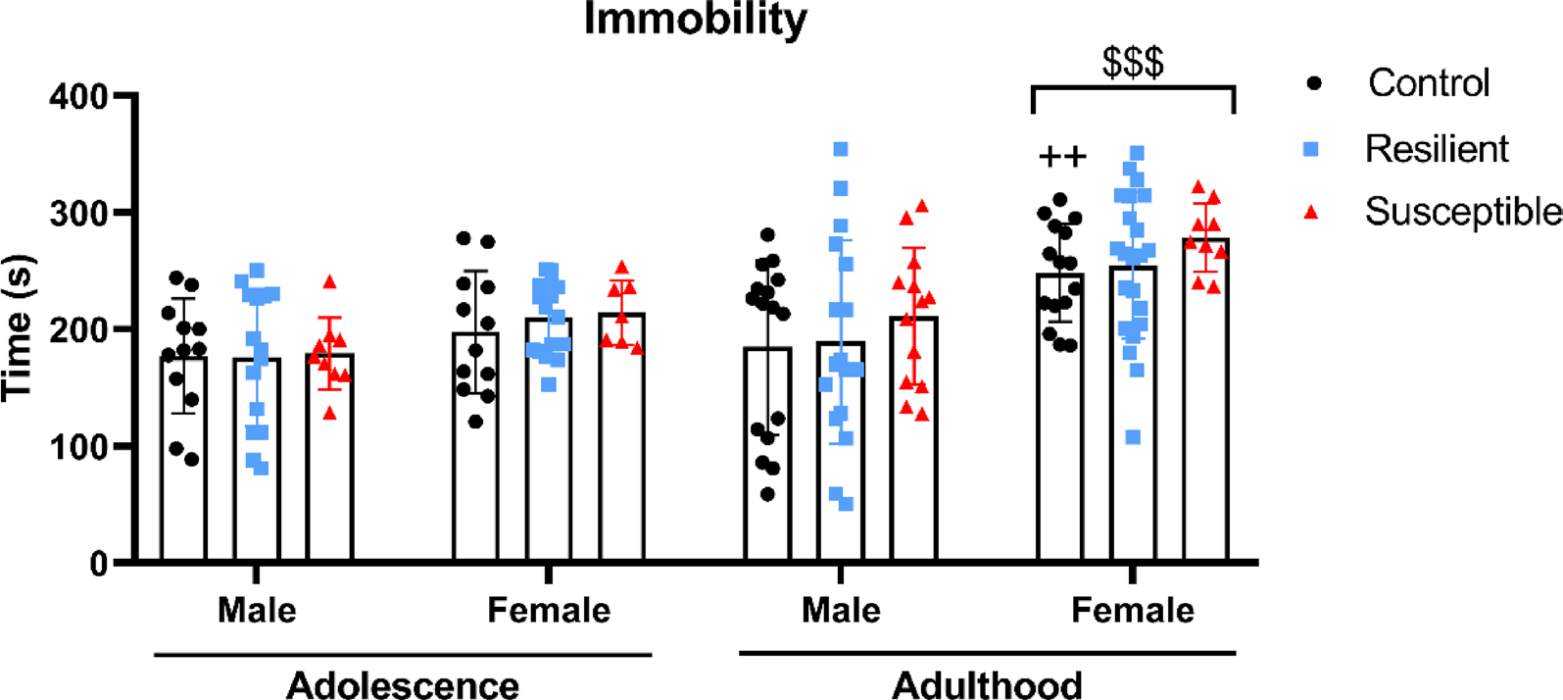
Effects of SD and VSD on the tail-suspension test in male and female mice. ++ *p* < 0.01 vs. their corresponding male groups; $$$ *p* < 0.001 vs. their corresponding adolescent groups

For the time in immobility, the ANOVA revealed no statistical differences (Fig. 6).

b) Long-term.

For the time in immobility, the ANOVA revealed an effect of the variable Sex [F [1, 29] = 8.372; *p* = 0.007]. Control adult female mice spent more time in immobility than control adult males (*p* < 0.01) (Fig. 6).

For the time in immobility, the ANOVA also revealed an effect of the variable Time [F [1, 76] = 23.295; *p* < 0.001] in female mice. Adult female mice spent more time in immobility than adolescent female animals (*p* < 0.001) (Fig. 6).

### Effect of social defeat on the brain KYN pathway

**Fig. 7:**
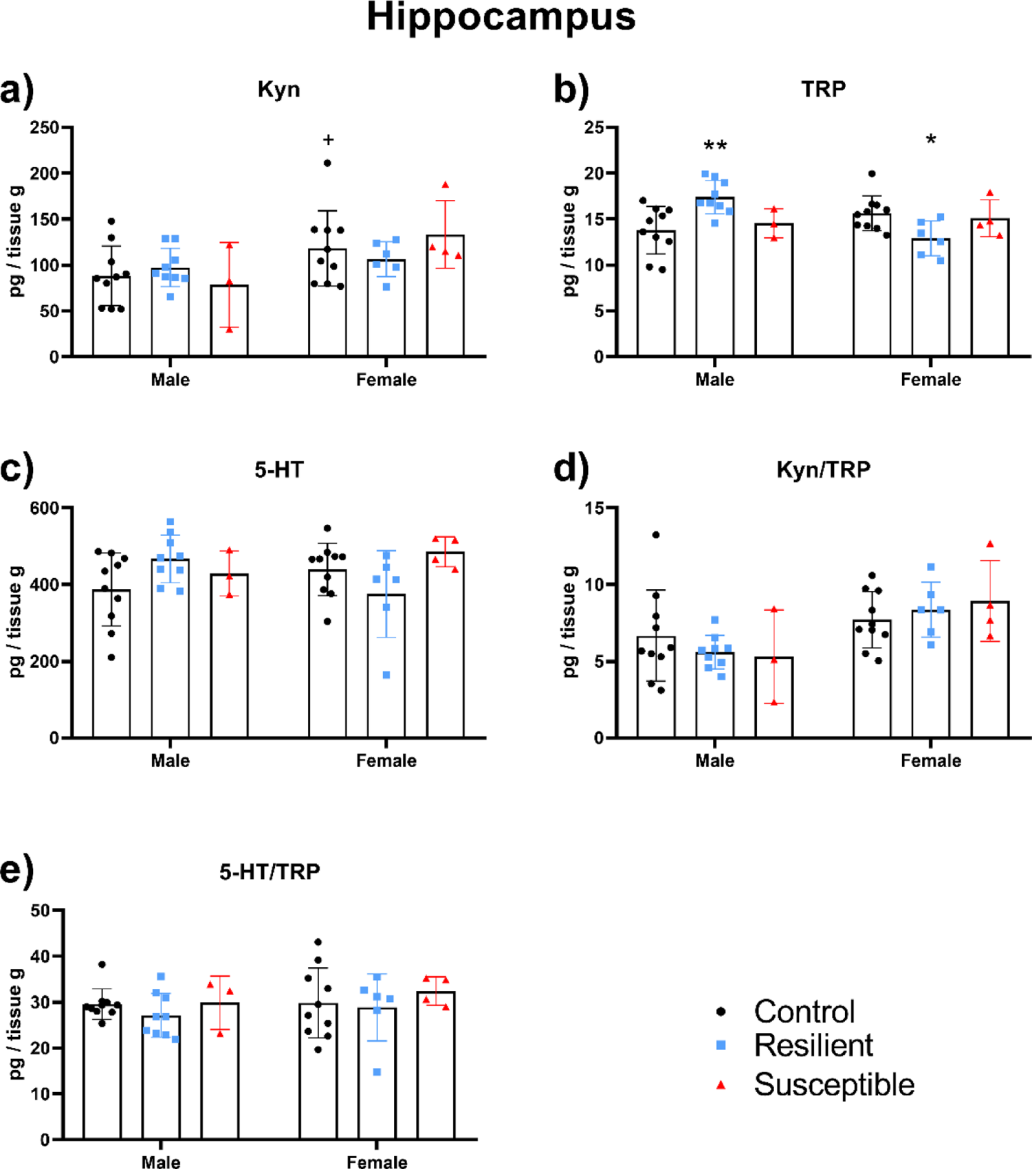
Alterations in the KYN pathway in the hippocampus of male and female mice after SD/VSD exposure. Graphs represent (**a**) kynurenine (KYN), (**b**) tryptophan (TRP), and (**c**) serotonin (5-HT) concentrations and (**d**) KYN/TRP and (**e**) 5-HT/TRP ratios. Results are shown as mean ± SEM (*n* = 3–10). Bonferroni post-hoc * *p* < 0.05, ** *p*  < 0.01 vs. the corresponding control group; +++ *p* < 0.001 vs. the corresponding male group

The KYN pathway was measured in the hippocampus, cerebellum, limbic forebrain, and striatum 24 h after the last social defeat stress. In females, the ANOVA showed an effect of stress for the hippocampal levels of tryptophan [F [2, 17] = 3.858; *p* = 0.042] and the striatal levels of kynurenine [F [2, 17] = 5.374; *p* = 0.016]. In both the hippocampus and the striatum, resilient defeated females showed lower tryptophan and KYN levels, respectively, than controls (*p* < 0.05 in both cases) (Figs. 7b and 10a).

**Fig. 8:**
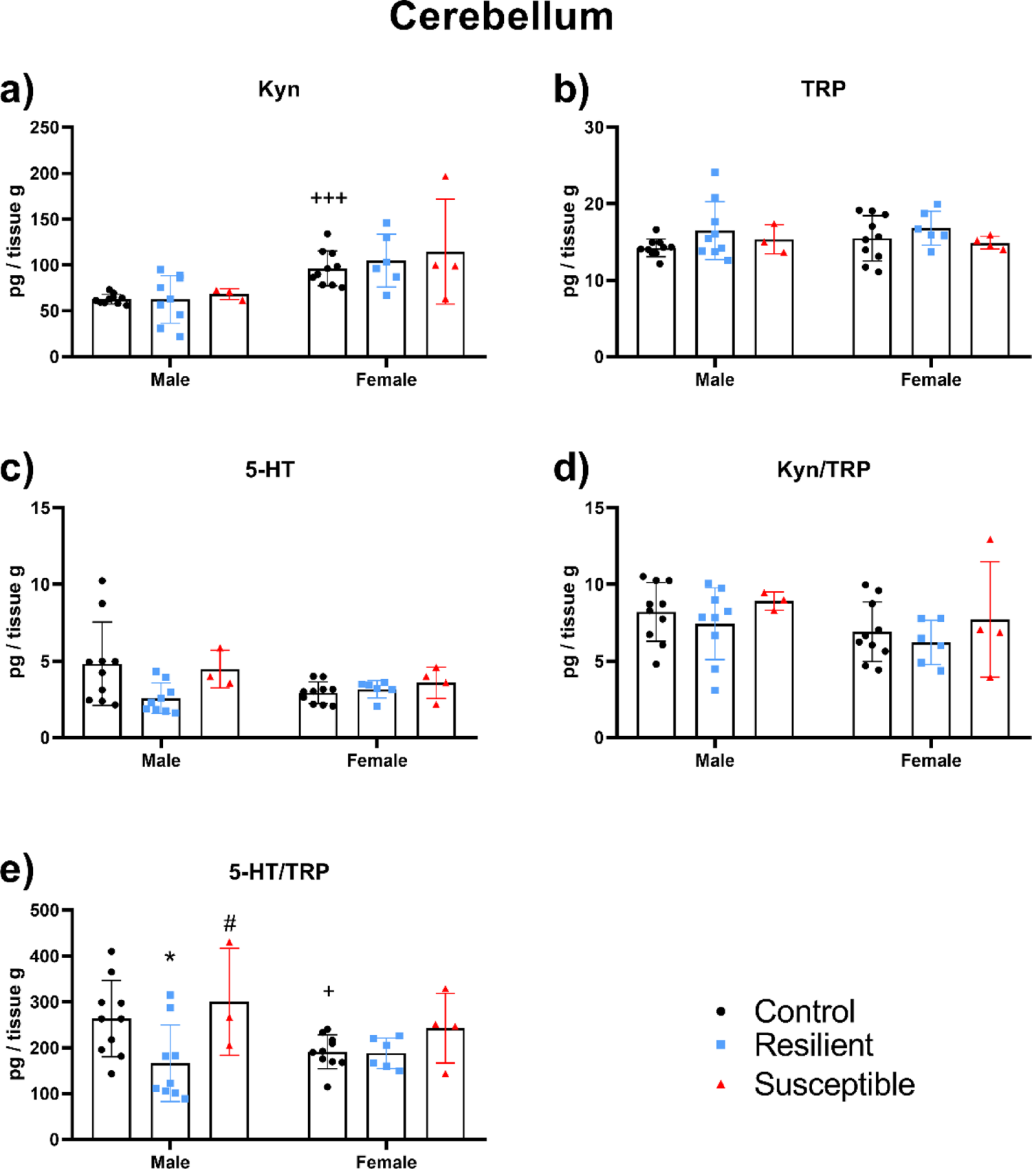
Alterations in the KYN pathway in the cerebellum of male and female mice after SD/VSD exposure. Graphs represent (**a**) kynurenine (KYN), (**b**) tryptophan (TRP), and (**c**) serotonin (5-HT) concentrations and (**d**) KYN/TRP and (**e**) 5-HT/TRP ratios. Results are shown as mean ± SEM (n=3–10). Bonferroni post-hoc *p <0.05 vs the corresponding control group; + *p* <0.05, +++ *p* <0.001 vs the corresponding male group; # *p* <0.05 vs the corresponding resilient group

**Fig. 9:**
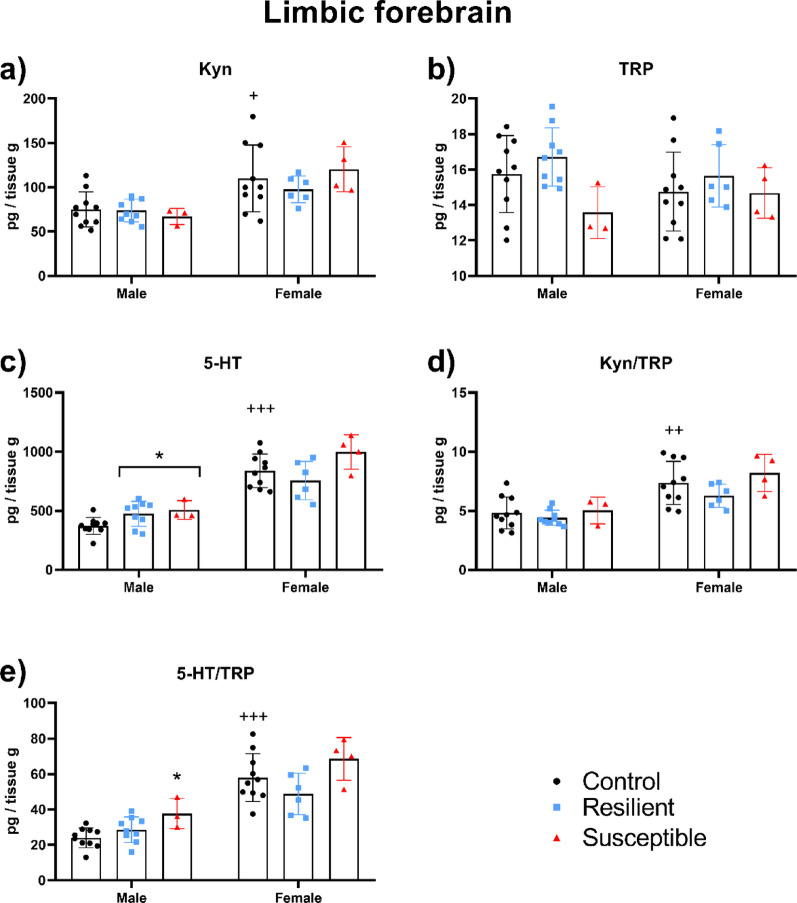
Alterations in the KYN pathway in the limbic forebrain of male and female mice after SD/VSD exposure. Graphs represent (**a**) kynurenine (KYN), (**b**) tryptophan (TRP), and (**c**) serotonin (5-HT) concentrations and (**d**) KYN/TRP and (**e**) 5-HT/TRP ratios. Results are shown as mean ± SEM (n=3–10). Bonferroni post-hoc * *p* <0.05 vs the corresponding control group; ++ *p*<0.01, +++ *p* <0.001 vs the corresponding male group

**Fig. 10:**
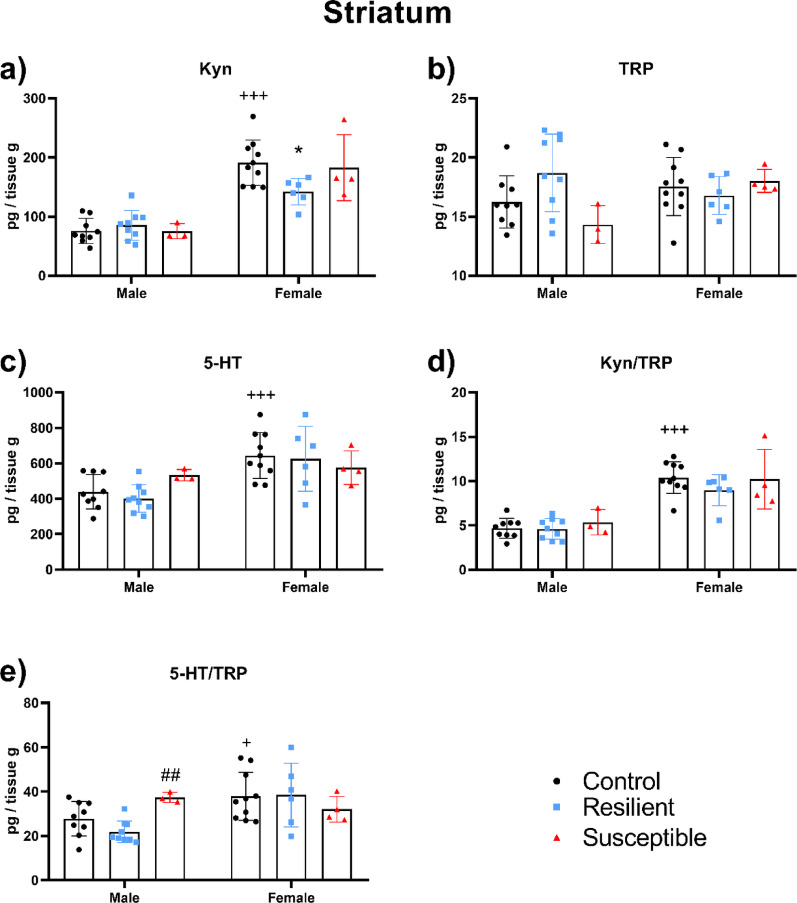
Alterations in the KYN pathway in the striatum of male and female mice after SD/VSD exposure. Graphs represent (**a**) kynurenine (KYN), (**b**) tryptophan (TRP), and (**c**) serotonin (5-HT) concentrations and (**d**) KYN/TRP and (**e**) 5-HT/TRP ratios. Results are shown as mean ± SEM (n=3–10). Bonferroni post-hoc * *p* <0.05 vs the corresponding control group; + *p* <0.05; +++ *p* <0.001 vs the corresponding male group; ## *p* <0.01 vs the corresponding resilient group

In males, the ANOVA revealed an effect of stress for the hippocampal levels of tryptophan [F [2, 18] = 7.212; *p* = 0.005], with resilient males showing higher tryptophan levels than control mice (*p* < 0.01) (Fig. 7b). In the limbic forebrain, the ANOVA showed an effect of stress for serotonin levels [F [2, 18] = 3.763; *p* = 0.043], with resilient and susceptible male mice showing higher serotonin levels than controls (*p* < 0.05 in both cases) (Fig. 10c). The ANOVA also showed an effect of stress for the serotonin/tryptophan ratio in the cerebellum [F [2, 18] = 6.003; *p* = 0.01], striatum [F [2, 18] = 7.428; *p* = 0.004], and limbic forebrain [F [2, 18] = 4.375; *p* = 0.028]. In the cerebellum, resilient male mice showed a lower ratio than controls and susceptible mice (*p* < 0.05 in both cases) (Fig. 8e). On the other hand, susceptible mice showed a higher ratio than resilient mice (*p* < 0.01) in the striatum (Fig. 10e), and than controls (*p* < 0.05) in the limbic forebrain (Fig. 9e).

For the differences among non-stressed control mice depending on sex, the ANOVA showed an effect for kynurenine levels in the hippocampus [F [1, 17] = 5.244; *p* = 0.035], cerebellum [F [1, 17] = 25.105; *p* = 0.001], limbic forebrain [F [1, 17] = 5.501; *p* = 0.031], and striatum [F [1, 17] = 63.695; *p* = 0.001]. For the KYN/tryptophan ratio, the ANOVA also showed an effect in the limbic forebrain [F [1, 17] = 10.211; *p* = 0.005] and striatum [F [1, 17] = 69.213; *p* = 0.001]. In all these structures, control female mice showed higher KYN levels and KYN/tryptophan ratios than males (*p* < 0.001 in all cases, except *p* < 0.05 for KYN levels in the hippocampus and limbic forebrain, and *p* < 0.01 for the KYN/tryptophan ratio in the limbic forebrain). The ANOVA also showed an effect for serotonin levels in the limbic forebrain [F [1, 17] = 76.285; *p* = 0.001] and striatum [F [1, 17] = 14.848; *p* = 0.001], with females showing higher serotonin levels than males (*p* < 0.001) (Figs. 9c and 10c). Furthermore, the ANOVA showed an effect for the serotonin/tryptophan ratio in the cerebellum [F [1, 17] = 5.211; *p* = 0.036], limbic forebrain [F [1, 17] = 47.608; *p* = 0.001], and striatum [F [1, 17] = 5.390; *p* = 0.033]. Control female mice showed a higher serotonin/tryptophan ratio in the limbic forebrain and striatum (*p* < 0.001 and *p* < 0.05, respectively) (Figs. 9e and 10e), but males showed a higher ratio in the cerebellum (*p* < 0.05) (Fig. 8e).

## Discussion

The most prevalent stressors in humans are psychological or social in nature, and the influence of stressful experiences on the emergence of psychopathological conditions has been extensively examined in preclinical animal research [43]. The SD model represents an ethologically relevant stressor that induces a broad range of molecular, physiological, and behavioral alterations similar to those observed in depression and anxiety [44–46]. SD has also been linked to substance use disorders [47] and increased neuroinflammatory responses [8, 48], and has been widely used to model the individual differences in stress responses observed in humans [49]. However, this model has been applied mainly to males, whereas the development of VSD has enabled the study of social conflict in females, avoiding physical harm while highlighting the psychosocial dimension of stress.

Strong evidence links exposure to traumatic events, particularly during early life, to an increased risk of developing mental illnesses later in life [50]. Similar to humans, adolescent rodents are highly social [51, 52], and studies conducted in adolescent defeated mice have reported reduced social behavior and increased depressive- and anxiety-like behaviors, as observed in defeated rodents during adulthood [16, 53, 54]. However, both in humans and animal models, only a proportion of individuals show vulnerability to stress-induced psychopathology. After exposure to SD, some mice display marked susceptibility, characterized by social withdrawal, anhedonia, and other depressive-like behaviors [55]. In contrast, other animals demonstrate resilience, successfully adapting to stressful experiences without developing maladaptive outcomes [56]. Although numerous studies have focused on resilient responses to social stress experienced during adulthood, very few have assessed this phenotype in animals stressed during adolescence, and studies in adolescent females are even more scarce.

Our results demonstrate that exposure to SD or VSD in adolescent male and female mice induces activation of the hypothalamic–pituitary–adrenal (HPA) axis, similarly to that observed in adult animals. Likewise, social stress during adolescence generates two populations of animals—resilient or susceptible to social withdrawal (a depressive-like behavior). Notably, vicarious exposure to SD results in a lower percentage of susceptible females than that observed in males. Importantly, we found that the behavioral and biochemical phenotypes of resilient and susceptible animals are clearly distinct, despite both groups showing similar increases in corticosterone levels. Regarding the persistence of stress-induced changes, our results indicate that these effects are primarily short-term, with stress-related alterations during adulthood largely restricted to increased anxiety in females. In the short-term, resilient and susceptible male mice traveled longer distances and at higher speeds than controls in the OF test, without vicariously stressed female mice showing changes. Only resilient mice showed increased anxiety-like behaviors, as assessed by the EPM, in both the short-term (males only) and the long-term (females only). Changes in anhedonia-related behaviors evaluated using the splash test were observed in defeated animals in the short-term, but with a marked sex difference. Defeated adolescent male animals showed an increase in time spent back grooming, whereas adolescent defeated females tended to decrease this grooming during this test.

With respect to alterations in the KYN pathway, exposure to social stress during adolescence decreased KYN levels only in the striatum of resilient female mice. Our results suggest that, exclusively in male mice, the serotonin/tryptophan ratio seems to be a good biochemical marker for a resilience/susceptibility profile. Susceptible males showed an increased serotonin/tryptophan ratio in the cerebellum, limbic forebrain, and striatum compared with their control or resilient counterparts.

### Social stress in adolescent mice induced depression and anxiety like behaviors

Our previous work has shown that defeated adult male mice classified as resilient to social avoidance (SIT test) exhibit a stable and consistent protective profile. These animals are not only protected against depressive-like effects of SD but also show reduced sensitivity to the reinforcing properties of drugs such as cocaine and alcohol [22, 34, 57]. In adolescent mice, however, the resilient response to SD appears to be more heterogeneous and complex. To date, only a limited number of studies have explored stress resilience during adolescence, mainly focused in male animals. More recent findings indicate that the proportion of adolescent male mice displaying resilience to depressive-like behaviors is comparable to that observed in adulthood [23]. It should be considered that male mice performance in the SIT may be influenced by the hierarchy established within the home cage, particularly in a highly territorial strain such as OF1. Interestingly, these resilient adolescent males exhibited heightened anxiety levels and showed an increased preference for cocaine and elevated ethanol intake. However, a neuroinflammatory response was primarily observed in the striatum of susceptible animals. Collectively, these results suggest that, unlike what has been described in adults, stress responses during adolescence are distinct and multifaceted. Special caution is required when interpreting and translating resilience-related phenotypes at this developmental stage, considering that most studies have been performed only in male subjects.

As previously reported, in the present study susceptible mice exhibited increased avoidance of contact with a novel mouse, as determined by the SIT. The percentage of susceptible animals was higher in males. These results are in line with those observed in defeated adult male and female mice. In previous studies a reduction in social behavior was not observed in females after being exposed to a similar VSD procedure [17, 19]. Although VSD effectively induces a stress response in females, it cannot be considered the same experience as SD. Likely, female mice are less stressed than males, as the physical component of the stress is absent. In fact, males not only physically undergo an attack but also lose territory that is motivationally relevant to them. One could consider that the smaller percentage of stressed females showing a decrease in social contact is due to a diminished stress experience, since they are only affected by “emotional contagion”. However, after being exposed to an accelerated social defeat stress in which females were physically attacked by an aggressive male twice a day for 4 days, Pantoja-Urbán and colleagues [58] did not observe a decrease in social interaction in most defeated females. These findings, together with our results, suggest that females show protection against stress-induced social avoidance, regardless of the type of social stress employed. Intriguingly, a recent report by Vanderhoof and colleagues [59] suggests that elevated levels of corticosterone after social stress could favor a resilient response. These authors observed that a single administration of corticosterone after exposure to SD during adolescence prevented defeat-induced social avoidance and anhedonia and potentiated morphine place preference in adult male mice.

Only defeated male mice showed a short-term increase in motor activity in the OF compared with their respective controls. Resilient and susceptible males covered more distance, with the susceptible mice also moving faster. These results agree with those of García-Pardo and colleagues [60] in mice defeated during adulthood and measured one week later. This increase in motor activity was not long-lasting, as no differences were observed in adult mice. Although SD/VSD did not induce changes in the time spent in the center of the open field, changes were dependent on the age of the animals. During adolescence, male mice spent more time in the center than adult male and female mice. Therefore, males decreased their time in the center with age, a decrease that was not observed in females.

Characteristically, SD increases anxiety in male mice defeated during adulthood [25, 61, 62], although results in females are less consistent, with increases or no changes reported in female mice exposed to VSD [17, 18]. However, few studies have evaluated the development of anxiety after SD during adolescence. Our results showed that shortly after exposure to SD, only resilient male mice exhibited elevated anxiety in the EPM, with a decrease in time spent, percentage of time, and percentage of entries into the open arms of the maze. However, susceptible mice showed no differences compared with non-stressed control mice. These data confirm previous results obtained in adolescent male mice [23]. In contrast, Vassilev and colleagues [25] reported a positive correlation between the SIT ratio and the percentage of time spent in the open arms of the EPM only in male mice exposed to an accelerated SD protocol during adolescence, but not during adulthood. Differences in the SD protocol employed could explain these divergent results. Our present findings extend this evidence by showing that the increase in anxiety in adulthood is only observed in resilient female mice. Although adolescent female mice did not show increased anxiety, adult females spent less time and a lower percentage of time in the open arms. In contrast, a recent study by Canto-de-Souza and colleagues [63] observed increased anxiety in both male and female adolescent mice after 10 days of vicarious exposure to SD. However, when comparing those results with the current study, we must take into consideration both the difference in number of stress sessions as well as the difference in when the response to stress and the EPM were measured. While the mice in Canto-de-Souza and colleagues’ study were exposed to VSD in late adolescence (PND 50 to 59) and the EPM was measured in adulthood (PND 61), our study tested the response to stress during the early adolescent period (PND 26 to 35) with EPM measured on PND 39.The results regarding stress-induced anxiety are heterogeneous and complex to interpret, mainly due to variations in the social stress procedure and the time elapsed between stress exposure and behavioral evaluation. All these methodological differences could explain the divergent results. For example, a recent study reported increased anxiety in adolescent female mice exposed to VSD after the last stress episode [64]. In contrast, using a similar protocol, we did not observe changes in adolescent male mice two weeks after the last SD episode in the EPM [11]. Unlike these studies where defeated mice were analyzed together, our results may unmask subtle stress effects by distinguishing between resilient and susceptible stress responses. We observed a specific and anxiogenic response in resilient adolescent male mice and highlight the need for particular caution when interpreting emerging anxiety effects in adolescent females.

Additionally, we tested depressive-like behavior using the splash test and the TST. In the splash test, a decrease in time spent grooming the back is considered indicative of reduced motivation and self-care behavior, which are characteristic of apathy. The effect of SD on this test is not fully consistent. Although in most studies social stress decreased back grooming, others failed to find any effect [64–66]. We surprisingly observed that defeated adolescent male mice showed an increase in back grooming shortly after SD, a behavior that was not observed in stressed females, which instead tended to decrease grooming. Therefore, socially defeated females expressed anhedonia, confirming the results of Ródenas and colleagues [18]. Interestingly, in the study by Canto-de-Souza and colleagues [63], in male and female mice vicariously exposed to SD, males spent more time in back grooming than females, with no stress-related effects.

An increase in immobility time in the TST is considered to represent feelings of despair [67]. No stress-related effects were observed in this test, but adult control non-stressed female mice showed more immobility time than male mice, indicating a higher tendency toward depression-like behavior in adulthood. The lack of stress effects in the TST is not surprising, as many studies have found no changes after only four episodes of SD [60], although increases [64] or decreases have been observed in some studies [65].

To sum up, socially defeated adolescent resilient male mice showed increased corticosterone levels, as well as elevated motor activity and anxiety during adolescence, although they maintained social contacts and did not exhibit anhedonia or despair-like behaviors. In susceptible males, the decrease in social contacts was accompanied by increased hyperlocomotion. Vicariously defeated adolescent female mice also showed increased corticosterone levels but maintained normal social and anxiety-related behavior, although an anxiogenic response emerged in adulthood. In resilient and susceptible females, a tendency toward anhedonia appeared during adolescence, accompanied by a sex-characteristic predisposition to despair-like behavior in adulthood.

### Social stress-induced changes in the KYN pathway in adolescent mice

Serotonin plays an important role in the regulation of stress responses and has been related to the pathophysiology of depression and anxiety [68, 69]. Stress induces increased serotonin release, synthesis, and turnover across multiple brain regions [70–72]. Although tryptophan serves as a precursor for serotonin synthesis, the KYN pathway accounts for approximately 95% of tryptophan metabolism [26]. In addition to modulating serotonergic neurotransmission, the KYN pathway also regulates glutamatergic neurotransmission [73, 74].

A growing body of evidence has linked alterations in the KYN pathway to inflammatory processes as well as to depression [75, 76]. A common finding in neurodegenerative and neuropsychiatric disorders is a marked reduction in serum tryptophan levels, due to enhanced tryptophan degradation through the KYN pathway. Increased proinflammatory cytokine release upregulates KYN enzymes, thereby linking immune activation to altered tryptophan metabolism and behavioral dysfunction [77]. According to this body of evidence, compelling studies confirm hyperactivity of the KYN pathway in stress-related disorders [78–80].

Ethical considerations required the use of a reduced number of animals for the study of the kynurenine pathway. This resulted in a small sample size in the susceptible groups of both males and females. It should be considered that potential differences with other groups may not have been detected in the statistical analysis due to this limitation. Our results showed that only resilient adolescent females exhibited a decrease in KYN levels in the striatum, with no changes observed in males. Notably, female mice, regardless of stress condition, presented higher KYN levels in all structures studied. To date, there are no conclusive studies reporting higher KYN levels in female animals, an effect consistently observed in our study. In a previous report performed in adult male mice, we showed that resilience and susceptibility to SD are related to the KYN pathway in the cerebellum. SD induced a long-lasting increase in KYN synthesis and in the KYN/5-HT ratio in adult defeated male mice, with this increase being significantly lower in those classified as resilient [34]. Similarly, other studies have also observed increased KYN levels in several brain regions after chronic social defeat in adult male mice [81, 82].

To date, few studies have evaluated the KYN response to stress in adolescents or in female animals. Similar to our findings, no changes in KYN levels were observed after a 20-day SD protocol applied to late adolescent male mice in the study by Câmara and Brandão [83]. Gracia-Rubio and colleagues [84] evaluated the impact of maternal separation during early adolescence in male and female mice and did not find any effects on KYN, serotonin, or tryptophan levels in the prefrontal cortex. A recent study by Nikkheslat and colleagues [85] suggests sex-specific alterations in the KYN pathway in adolescent boys and girls with a diagnosis of depression. The authors reported that adolescents at high risk for depression or with major depressive disorder presented lower kynurenic acid concentrations and lower kynurenic acid/quinolinic acid ratios than low-risk adolescents. Interestingly, these differences were not observed in boys but were driven by the female results. Female individuals with persistent major depressive disorder at the 3-year follow-up showed lower baseline KYN levels and higher 3-hydroxykynurenine/KYN ratios than those who experienced remission at follow-up. Although these studies were either performed in humans or, in the case of animal studies in adult subjects, and therefore, are not fully comparable to the present design, they generally suggest that stress responses in adolescent males do not correlate with KYN levels, whereas decreases in KYN in females are associated with more stress-related symptoms.

In our study, we also observed a sex-dependent effect in the kynurenine to tryptophan ratio (KYN/TRP) with higher levels in the limbic brain and striatum in control adolescent females compared with adolescent males. The KYN/TRP ratio is widely used as an indicator of kynurenine pathway activation, reflecting the enzymatic conversion of tryptophan into kynurenine, a process that is often upregulated under conditions of stress and inflammation [86–88]. The higher KYN/TRP ratio observed in females is particularly relevant, as activation of the KYN pathway has been associated with increased vulnerability to anxiety- and depression-like behaviors, especially in the context of chronic stress or social defeat paradigms [89]. No direct comparison between defeated male and female mice can be performed due to the different protocols employed to induce social stress. However, these differences in non-stressed mice agree with previous studies indicating that females are generally more susceptible to the behavioral and neuroendocrine consequences of social stress, frequently exhibiting stronger inflammatory responses and sex-specific metabolic alterations [90–92]. In this context, sex differences in the KYN/TRP ratio may reflect underlying neurobiological and endocrine mechanisms, including greater sensitivity of the HPA axis and enhanced immune signaling in females [93]. Consistent with these neurochemical findings, our behavioral analyses revealed that resilient and susceptible adolescent females spent less time engaged in back grooming than both control animals and adolescent males. Reduced self-grooming behavior is commonly interpreted as a sign of apathy or reduced motivation and is often considered a behavioral correlate of depressive-like states in rodent models. Taken together, the elevated KYN/TRP ratio observed in females suggests a greater vulnerability to stress-induced emotional disturbances compared with males. These findings align with previously reported patterns of stress susceptibility in both animal models and humans and may contribute to explaining the higher prevalence of affective disorders observed in women.

A common finding in male mice observed in the cerebellum, limbic forebrain, and striatum is that susceptible mice presented a higher serotonin/tryptophan ratio compared with resilient or control animals. These results suggest that the biochemical signature of resilience or susceptibility after an adolescent stress experience could be related to changes in the relative levels of serotonin and tryptophan in these brain structures. We previously observed comparable changes in the cerebellum of adult defeated male mice, with a tendency toward an increased ratio in susceptible versus resilient animals [34]. An elegant study by Prakash and colleagues [94] in adult male rats reported an increased number of neurons expressing serotonin biosynthetic enzymes in the dorsal raphe nucleus in susceptible animals after prolonged exposure to SD and intracranial self-stimulation. In that study, susceptibility to SD was defined based on an anhedonia response reflected by an increased intracranial self-stimulation threshold. Despite methodological differences, these results and ours suggest that the resilient phenotype in stressed animals may be related to lower serotonin levels relative to tryptophan. However, it should be noted that although adolescent resilient mice did not show reduced social contact, they did exhibit increased anxiety in contrast to adult resilient mice, which maintain a fully resilient behavioral and neuroimmune profile. Finally, we observed that resilience induced opposite changes in hippocampal tryptophan levels, with resilient males showing an increase but resilient females a decrease compared to controls. Previous results in adult male mice reported a decrease in tryptophan cerebellar levels in both resilient and susceptible defeated males [34]. To sum up, our results suggest that social stress induces more changes in the KYN pathway and in more brain areas during adolescence than in adult animals. More changes were evident in male animals exposed to SD compared with the vicarious experience in females.

## Conclusions

This study highlights the particular response of adolescent animals to social stress by comparing the classical SD protocol in males with the vicarious experience in female mice. We confirm that both protocols induce behavioral effects and changes in the KYN pathway, with only resilient female mice showing a decrease in KYN levels in the striatum. In summary, the resilient phenotype in defeated animals during adolescence is not consistent, as anxiety is observed in animals resilient to social avoidance, whereas diminished changes were detected in susceptible ones. The development of resilience/susceptibility to social stress in males seems to be linked to a decrease in serotonin levels in the cerebellum and limbic forebrain relative to tryptophan. Therefore, an increase in the 5-HT/TRP ratio in susceptible male mice was associated with social avoidance but not with increased anxiety. Understanding the mechanistic contribution of the KYN pathway to the pathophysiology of mental diseases would have important translational benefits.

## Data Availability

The datasets used and/or analyzed during the current study are available from the corresponding author on reasonable request.

## References

[1] Copeland WE, Shanahan L, Hinesley J, Chan RF, Aberg KA, Fairbank JA, et al. Association of Childhood Trauma Exposure With Adult Psychiatric Disorders and Functional Outcomes. JAMA Netw Open. 2018;1(7). 10.1001/jamanetworkopen.2018.4493 . 10.1001/jamanetworkopen.2018.4493 PMC6324370 30646356

[2] Smith KE, Pollak SD. Early life stress and development: potential mechanisms for adverse outcomes. J Neurodev Disord. 2020;12(1). 10.1186/s11689-020-. 10.1186/s11689-020-09337-y PMC7745388 33327939

[3] Suglia SF, Koenen KC, Boynton-Jarrett R, Chan PS, Clark CJ, Danese A, et al. Childhood and Adolescent Adversity and Cardiometabolic Outcomes: A Scientific Statement From the American Heart Association. Circulation. 2018;137(5):e15–28. 10.1161/CIR.0000000000000536. 29254928 10.1161/CIR.0000000000000536 PMC7792566

[4] Leconte C, Mongeau R, Noble F. Traumatic Stress-Induced Vulnerability to Addiction: Critical Role of the Dynorphin/Kappa Opioid Receptor System. Front Pharmacol. 2022;13. 10.3389/fphar.2022.856672 . 10.3389/fphar.2022.856672 PMC9091501 35571111

[5] Gerhardt C, Semmer NK, Sauter S, Walker A, de Wijn N, Kälin W, et al. How are social stressors at work related to well-being and health? A systematic review and meta-analysis. BMC Public Health. 2021;21(1). 10.1186/s12889-021-10894-7 . 10.1186/s12889-021-10894-7 PMC8111761 33971850

[6] Miczek KA. A new test for aggression in rats without aversive stimulation: differential effects of d-amphetamine and cocaine. Psychopharmacology. 1979;60(3):253–9. 10.1007/BF00426664. 108702 10.1007/BF00426664

[7] Shimamoto A. Social Defeat Stress, Sex, and Addiction-Like Behaviors. Int Rev Neurobiol. 2018;140:271–313. 10.1016/bs.irn.2018.07. 30193707 10.1016/bs.irn.2018.07.009

[8] Montagud-Romero S, Blanco-Gandía MC, Reguilón MD, Ferrer-Pérez C, Ballestín R, Miñarro J, et al. Social defeat stress: Mechanisms underlying the increase in rewarding effects of drugs of abuse. Eur J Neurosci. 2018;48(9):2948–70. 10.1111/ejn.14127 . 30144331 10.1111/ejn.14127

[9] Nestler EJ, Russo SJ. Neurobiological basis of stress resilience. Neuron. 2024;112(12):1911–29. 10.1016/j.neuron.2024.05. 38795707 10.1016/j.neuron.2024.05.001 PMC11189737

[10] Han X, DeBold JF, Miczek KA. Prevention and reversal of social stress-escalated cocaine self-administration in mice by intra-VTA CRFR1 antagonism. Psychopharmacology. 2017;234(18):2813–21. 10.1007/s00213-017-4676-8 . 28698920 10.1007/s00213-017-4676-8 PMC5709170

[11] Rodriguez-Arias M, Navarrete F, Blanco-Gandia MC, Arenas MC, Bartoll-Andrés A, Aguilar MA, et al. Social defeat in adolescent mice increases vulnerability to alcohol consumption. Addict Biol. 2016;21(1):87–97. 10.1111/adb.12184 . 25219790 10.1111/adb.12184

[12] Kuske JX, Trainor BC. Mean Girls: Social Stress Models for Female Rodents. Curr Top Behav Neurosci. 2022;54:95–124. 10.1007/7854_2021_247. 34532840 10.1007/7854_2021_247 PMC9812211

[13] Sial OK, Warren BL, Alcantara LF, Parise EM, Bolaños-Guzmán CA. Vicarious social defeat stress: Bridging the gap between physical and emotional stress. J Neurosci Methods. 2016;258:94–103. 10.1016/j.jneumeth.2015.10.012. 26545443 10.1016/j.jneumeth.2015.10.012 PMC4691556

[14] Warren BL, Vialou VF, Iñiguez SD, Alcantara LF, Wright KN, Feng J, et al. Neurobiological sequelae of witnessing stressful events in adult mice. Biol Psychiatry. 2013;73(1):7–14. 10.1016/j.biopsych.2012.06.006. 22795644 10.1016/j.biopsych.2012.06.006 PMC3498570

[15] Carnevali L, Montano N, Tobaldini E, Thayer JF, Sgoifo A. The contagion of social defeat stress: Insights from rodent studies. Neurosci Biobehav Rev. 2020;111:12–8. 10.1016/j.neubiorev.2020.01.011. 31931035 10.1016/j.neubiorev.2020.01.011

[16] Iñiguez SD, Riggs LM, Nieto SJ, Dayrit G, Zamora NN, Shawhan KL, et al. Social defeat stress induces a depression-like phenotype in adolescent male c57BL/6 mice. Stress. 2014;17(3):247–55. 10.3109/10253890.2014.910650 . 24689732 10.3109/10253890.2014.910650 PMC5534169

[17] González-Portilla M, Montagud-Romero S, Rodríguez de Fonseca F, Rodríguez-Arias M. Oleoylethanolamide restores stress-induced prepulse inhibition deficits and modulates inflammatory signaling in a sex-dependent manner. Psychopharmacology. 2023;242(5):913. 10.1007/s00213-023-06403-w. 37314479 10.1007/s00213-023-06403-w PMC12043760

[18] Ródenas-González F, Arenas MC, Blanco-Gandía MC, Manzanedo C, Rodríguez-Arias M. Vicarious Social Defeat Increases Conditioned Rewarding Effects of Cocaine and Ethanol Intake in Female Mice. Biomedicines. 2023;11(2). 10.3390/biomedicines11020502. 10.3390/biomedicines11020502 PMC9953170 36831038

[19] Torres-Rubio L, Reguilón MD, Mellado S, Pascual M, Rodríguez-Arias M. Effects of Ketogenic Diet on Increased Ethanol Consumption Induced by Social Stress in Female Mice. Nutrients. 2024;16(17). 10.3390/nu16172814. 10.3390/nu16172814 PMC11397041 39275131

[20] File SE. The use of social interaction as a method for detecting anxiolytic activity of chlordiazepoxide-like drugs. J Neurosci Methods. 1980;2(3):219–38. 10.1016/0165-0270. 6120260 10.1016/0165-0270(80)90012-6

[21] File SE, Seth P. A review of 25 years of the social interaction test. Eur J Pharmacol. 2003;463(1–3):35–53. 10.1016/S0014-2999(03)01273-1. 12600701 10.1016/s0014-2999(03)01273-1

[22] Ballestín R, Alegre-Zurano L, Ferrer-Pérez C, Cantacorps L, Miñarro J, Valverde O, et al. Neuroinflammatory and behavioral susceptibility profile of mice exposed to social stress towards cocaine effects. Prog Neuropsychopharmacol Biol Psychiatry. 2021;105. 10.1016/j.pnpbp.2020.110123. 10.1016/j.pnpbp.2020.110123 33002518

[23] Reguilón MD, Ballestín R, Miñarro J, Rodríguez-Arias M. Resilience to social defeat stress in adolescent male mice. Prog Neuropsychopharmacol Biol Psychiatry. 2022;119. 10.1016/j.pnpbp.2022.110591. 10.1016/j.pnpbp.2022.110591 35697171

[24] Alves-dos-Santos L, Resende L, de Chiavegatto S. Susceptibility and resilience to chronic social defeat stress in adolescent male mice: No correlation between social avoidance and sucrose preference. Neurobiol Stress. 2020;12:100221. 10.1016/j.ynstr.2020.100221. 32435670 10.1016/j.ynstr.2020.100221 PMC7231980

[25] Vassilev P, Pantoja-Urban AH, Giroux M, Nouel D, Hernandez G, Orsini T, et al. Unique effects of social defeat stress in adolescent male mice on the Netrin-1/DCC pathway, prefrontal cortex dopamine and cognition (Social stress in adolescent vs. adult male mice). eNeuro. 2021;8(2). 10.1523/ENEURO.0045-21. 10.1523/ENEURO.0045-21.2021 PMC8051112 33619036

[26] Schwarcz R. Metabolism and function of brain kynurenines. Biochem Soc Trans. 1993;21(1):77–82. 10.1042/bst.0210077. 8449358 10.1042/bst0210077

[27] Shi C, Dong J, Hui X, Xu Z, Zhao Z, Dong L. Production, Mechanisms, and Therapeutic Strategies of Tryptophan Metabolites in CNS Diseases. Mol Neurobiol. 2025;63(1). 10.1007/s12035-025-05538-5. 10.1007/s12035-025-05538-5 PMC12669377 41324859

[28] Campbell BM, Charych E, Lee AW, Möller T. Kynurenines in CNS disease: regulation by inflammatory cytokines. Front Neurosci. 2014;8(8 FEB). 10.3389/fnins.2014.00012. 10.3389/fnins.2014.00012 PMC3915289 24567701

[29] Gibney SM, McGuinness B, Prendergast C, Harkin A, Connor TJ, Poly I. C-induced activation of the immune response is accompanied by depression and anxiety-like behaviours, kynurenine pathway activation and reduced BDNF expression. Brain Behav Immun. 2013;28:170–81. 23201589 10.1016/j.bbi.2012.11.010

[30] Liu XC, Holtze M, Powell SB, Terrando N, Larsson MK, Persson A, et al. Behavioral disturbances in adult mice following neonatal virus infection or kynurenine treatment - Role of brain kynurenic acid. Brain Behav Immun. 2014;36:80–9. 10.1016/j.bbi.2013.10.010. 24140727 10.1016/j.bbi.2013.10.010 PMC3947209

[31] Larkin PB, Sathyasaikumar KV, Notarangelo FM, Funakoshi H, Nakamura T, Schwarcz R, et al. Tryptophan 2,3-dioxygenase and indoleamine 2,3-dioxygenase 1 make separate, tissue-specific contributions to basal and inflammation-induced kynurenine pathway metabolism in mice. Biochim Biophys Acta Gen Subj. 2016;1860(11):2345–54. 10.1016/j.bbagen.2016.07. 10.1016/j.bbagen.2016.07.002 PMC5808460 27392942

[32] Bay-Richter C, Janelidze S, Sauro A, Bucala R, Lipton J, Deierborg T, et al. Behavioural and neurobiological consequences of macrophage migration inhibitory factor gene deletion in mice. J Neuroinflammation. 2015;12(1). 10.1186/s12974-015-0387-4. 10.1186/s12974-015-0387-4 PMC4558780 26338025

[33] Setoyama D, Yoshino A, Takamura M, Okada G, Iwata M, Tsunetomi K, et al. Personality classification enhances blood metabolome analysis and biotyping for major depressive disorders: two-species investigation. J Affect Disord. 2021;279:20–30. 10.1016/j.jad.2020.09.118. 33038697 10.1016/j.jad.2020.09.118

[34] Giménez-Gómez P, Ballestín R, Gil de Biedma-Elduayen L, Vidal R, Ferrer-Pérez C, Reguilón MD, et al. Decreased kynurenine pathway potentiate resilience to social defeat effect on cocaine reward. Neuropharmacology. 2021;197. 10.1016/j.neuropharm.2021.108753. 10.1016/j.neuropharm.2021.108753 34389399

[35] Rodríguez-Arias M, Miñarro J, Aguilar MA, Pinazo J, Simón VM. Effects of risperidone and SCH 23390 on isolation-induced aggression in male mice. Eur Neuropsychopharmacol. 1998;8(2):95–103. 10.1016/S0924-977X. 9619687 10.1016/s0924-977x(97)00051-5

[36] Tornatzky W, Miczek KA. Long-term impairment of autonomic circadian rhythms after brief intermittent social stress. Physiol Behav. 1993;53(5):983–93. 10.1016/0031-9384(93)90278-N. 8511216 10.1016/0031-9384(93)90278-n

[37] Covington HE, Miczek KA. Repeated social-defeat stress, cocaine or morphine. Effects on behavioral sensitization and intravenous cocaine self-administration binges. Psychopharmacology. 2001;158(4):388–98. 10.1007/s002130100858. 11797060 10.1007/s002130100858

[38] Miczek KA, Thompson ML, Shuster L. Opioid-like analgesia in defeated mice. Science. 1982;215(4539):1520–2. 10.1126/science.7199758. 7199758 10.1126/science.7199758

[39] Berton O, McClung CA, DiLeone RJ, Krishnan V, Renthal W, Russo SJ, et al. Essential role of BDNF in the mesolimbic dopamine pathway in social defeat stress. Science. 2006;311(5762):864–8. 10.1126/science.1120972. 16469931 10.1126/science.1120972

[40] Henriques-Alves AM, Queiroz CM. Ethological Evaluation of the Effects of Social Defeat Stress in Mice: Beyond the Social Interaction Ratio. Front Behav Neurosci. 2016;9. 10.3389/fnbeh.2015.00364. 10.3389/fnbeh.2015.00364 PMC4737906 26869895

[41] Hodes GE, Pfau ML, Purushothaman I, Francisca Ahn H, Golden SA, Christoffel DJ, et al. Sex Differences in Nucleus Accumbens Transcriptome Profiles Associated with Susceptibility versus Resilience to Subchronic Variable Stress. J Neurosci. 2015;35(50):16362–76. 10.1523/JNEUROSCI.1392-15. 26674863 10.1523/JNEUROSCI.1392-15.2015 PMC4679819

[42] Golden SA, Covington HE, Berton O, Russo SJ. A standardized protocol for repeated social defeat stress in mice. Nat Protoc. 2011;6(8):1183–91. 10.1038/nprot.2011.361. 21799487 10.1038/nprot.2011.361 PMC3220278

[43] Björkqvist K. Social defeat as a stressor in humans. Physiol Behav. 2001;73(3):435–42. 10.1016/S0031-9384(01)00490-5. 11438372 10.1016/s0031-9384(01)00490-5

[44] Rygula R, Abumaria N, Flügge G, Fuchs E, Rüther E, Havemann-Reinecke U. Anhedonia and motivational deficits in rats: Impact of chronic social stress. Behav Brain Res. 2005;162(1):127–34. 10.1016/j.bbr.2005.03.009. 15922073 10.1016/j.bbr.2005.03.009

[45] Moore NLT, Altman DE, Gauchan S, Genovese RF. Adulthood stress responses in rats are variably altered as a factor of adolescent stress exposure. Stress. 2016;19(3):295–302. 10.1080/10253890.2016.1191465. 27295201 10.1080/10253890.2016.1191465

[46] Kinsey SG, Bailey MT, Sheridan JF, Padgett DA, Avitsur R. Repeated social defeat causes increased anxiety-like behavior and alters splenocyte function in C57BL/6 and CD-1 mice. Brain Behav Immun. 2007;21(4):458–66. 17178210 10.1016/j.bbi.2006.11.001 PMC1941837

[47] Miczek KA, Yap JJ, Covington HE. Social stress, therapeutics and drug abuse: Preclinical models of escalated and depressed intake. Pharmacol Ther. 2008;120(2):102–28. 10.1016/j.pharmthera.2008.07.006. 18789966 10.1016/j.pharmthera.2008.07.006 PMC2713609

[48] Stein DJ, Vasconcelos MF, Albrechet-Souza L, Ceresér KMM, De Almeida RMM. Microglial Over-Activation by Social Defeat Stress Contributes to Anxiety- and Depressive-Like Behaviors. Front Behav Neurosci. 2017;11. 10.3389/fnbeh.2017.00207. 10.3389/fnbeh.2017.00207 PMC5660717 29114211

[49] Wang W, Liu W, Duan D, Bai H, Wang Z, Xing Y. Chronic social defeat stress mouse model: Current view on its behavioral deficits and modifications. Behav Neurosci. 2021;135(3). 10.1037/bne0000418. 10.1037/bne0000418 33090813

[50] McLaughlin KA, Green JG, Gruber MJ, Sampson NA, Zaslavsky AM, Kessler RC. Childhood adversities and first onset of psychiatric disorders in a national sample of US adolescents. Arch Gen Psychiatry. 2012;69(11):1151–60. 10.1001/archgenpsychiatry.2011.2277. 23117636 10.1001/archgenpsychiatry.2011.2277 PMC3490224

[51] Ribeiro Do Couto B, Aguilar MA, Lluch J, Rodríguez-Arias M, Miñarro J. Social experiences affect reinstatement of cocaine-induced place preference in mice. Psychopharmacology. 2009;207(3):485–98. 10.1007/s00213-009-1678-1. 19798482 10.1007/s00213-009-1678-1

[52] Yates JR, Beckmann JS, Meyer AC, Bardo MT. Concurrent choice for social interaction and amphetamine using conditioned place preference in rats: Effects of age and housing condition. Drug Alcohol Depend. 2013;129(3):240–6. 23540449 10.1016/j.drugalcdep.2013.02.024 PMC3628407

[53] Huang GB, Zhao T, Muna SS, Bagalkot TR, Jin HM, Chae HJ, et al. Effects of chronic social defeat stress on behaviour, endoplasmic reticulum proteins and choline acetyltransferase in adolescent mice. Int J Neuropsychopharmacol. 2013;16(7):1635–47. 10.1017/S1461145713000060. 23442729 10.1017/S1461145713000060

[54] Shimizu T, Ishida A, Hagiwara M, Ueda Y, Hattori A, Tajiri N, et al. Social Defeat Stress in Adolescent Mice Induces Depressive-like Behaviors with Reduced Oligodendrogenesis. Neuroscience. 2020;443:218–32. 10.1016/j.neuroscience. 32652175 10.1016/j.neuroscience.2020.07.002

[55] Krishnan V, Han MH, Graham DL, Berton O, Renthal W, Russo SJ, et al. Molecular Adaptations Underlying Susceptibility and Resistance to Social Defeat in Brain Reward Regions. Cell. 2007;131(2):391–404. 10.1016/j..2007.09.018cell. 17956738 10.1016/j.cell.2007.09.018

[56] Cathomas F, Murrough JW, Nestler EJ, Han MH, Russo SJ. Neurobiology of Resilience: Interface Between Mind and Body. Biol Psychiatry. 2019;86(6):410–20. 10.1016/j.biopsych.201904.011. 31178098 10.1016/j.biopsych.2019.04.011 PMC6717018

[57] Reguilón MD, Ferrer-Pérez C, Manzanedo C, Miñarro J, Rodríguez-Arias M. Ethanol intake in male mice exposed to social defeat: Environmental enrichment potentiates resilience. Neurobiol Stress. 2021;15. 10.1016/j.ynstr.2021.100413. 10.1016/j.ynstr.2021.100413 PMC8591477 34815986

[58] Pantoja-Urbán AH, Richer S, Mittermaier A, Giroux M, Nouel D, Hernandez G, et al. Gains and Losses: Resilience to Social Defeat Stress in Adolescent Female Mice. Biol Psychiatry. 2024;95(1):37–47. 10.1016/j.biopsych.2023.06.014. 37355003 10.1016/j.biopsych.2023.06.014 PMC10996362

[59] Vanderhoof SO, Vincent CJ, Beaver JN, Latsko MS, Aguilar-Alvarez R, Jasnow AM. Corticosterone after early adolescent stress prevents social avoidance, aversive behavior, and morphine-conditioned place preference in adulthood. Psychopharmacology. 2024;241(10):2045–59. 10.1007/s00213-024-06616-7. 38805040 10.1007/s00213-024-06616-7 PMC11442498

[60] García-Pardo MP, Roger-Sánchez C, Rodríguez-Arias M, Miñarro J, Aguilar MA. Cognitive and behavioural effects induced by social stress plus MDMA administration in mice. Behav Brain Res. 2017;319:63–72. 27840246 10.1016/j.bbr.2016.11.012

[61] Ferrer-Pérez C, Reguilón MD, Manzanedo C, Miñarro J, Rodríguez-Arias M. Social Housing Conditions Modulate the Long-Lasting Increase in Cocaine Reward Induced by Intermittent Social Defeat. Front Behav Neurosci. 2019;13. 10.3389/fnbeh.2019.00148. 10.3389/fnbeh.2019.00148 PMC6622358 31333427

[62] Rodríguez-Arias M, Malaguarnera M, Ribes-Catalá M, Ferrer-Pérez C, Pascual M. Effects of environmental enrichment during adolescence on social defeat effects. Behav Neurosci. 2026;140(1). 10.1037/bne0000640. 10.1037/bne0000640 41411042

[63] Canto-de-Souza L, Baptista-de-Souza D, Thiele M, Garcia VG, Silva KC, de Souza FV, et al. Sex differences in behavioral and neural responses induced by witnessing social defeat stress during adolescence or adulthood in mice. Prog Neuropsychopharmacol Biol Psychiatry. 2025;138. 10.1016/j.pnpbp.2025.111313. 10.1016/j.pnpbp.2025.111313 40049344

[64] Martínez-Caballero MÁ, Calpe-López C, García-Pardo MP, Arenas MC, Manzanedo C, Aguilar MA. Voluntary wheel running prevented the short-term behavioural effects of vicarious intermittent social defeat in female mice. Psychopharmacology. 2025. 10.1007/s00213-025-06927-3. 41068505 10.1007/s00213-025-06927-3 PMC13506583

[65] Calpe-López C, García-Pardo MP, Martínez-Caballero MA, Santos-Ortíz A, Aguilar MA. Behavioral Traits Associated With Resilience to the Effects of Repeated Social Defeat on Cocaine-Induced Conditioned Place Preference in Mice. Front Behav Neurosci. 2020;13. 10.3389/fnbeh.2019.00278. 10.3389/fnbeh.2019.00278 PMC6962131 31998090

[66] Calpe-López C, Martínez-Caballero MA, García-Pardo MP, Aguilar MA. Intermittent voluntary wheel running promotes resilience to the negative consequences of repeated social defeat in mice. Physiol Behav. 2022;254. 10.1016/j.physbeh.2022.113916. 10.1016/j.physbeh.2022.113916 35850205

[67] Becker M, Pinhasov A, Ornoy A. Animal Models of Depression: What Can They Teach Us about the Human Disease? Diagnostics (Basel). 2021;11(1). 10.3390/diagnostics11010123. 10.3390/diagnostics11010123 PMC7830961 33466814

[68] Chen M, Wang C, Lin Y, Chen Y, Xie W, Huang X, et al. Dorsal raphe nucleus-hippocampus serotonergic circuit underlies the depressive and cognitive impairments in 5×FAD male mice. Transl Neurodegener. 2024;13(1). 10.1186/s40035-024-00425-w. 10.1186/s40035-024-00425-w PMC11267773 39044270

[69] Vahid-Ansari F, Newman-Tancredi A, Fuentes-Alvarenga AF, Daigle M, Albert PR. Rapid reorganization of serotonin projections and antidepressant response to 5-HT1A-biased agonist NLX-101 in fluoxetine-resistant cF1ko mice. Neuropharmacology. 2024;261. 10.1016/j.neuropharm.2024.110132. 10.1016/j.neuropharm.2024.110132 39208980

[70] Chaouloff F, Berton O, Mormède P. Serotonin and stress. Neuropsychopharmacology. 1999;21(2 Suppl):S28–32. 10.1016/S0893-133X.(99)00008-1. 10.1016/S0893-133X(99)00008-1 10432486

[71] Dunn J. Sibling influences on childhood development. J Child Psychol Psychiatry. 1988;29(2):119–27. 10.1111/j.1469-7610.1988.tb00697.x. 3286666 10.1111/j.1469-7610.1988.tb00697.x

[72] Al-Kachak A, Di Salvo G, Fulton SL, Chan JC, Farrelly LA, Lepack AE, et al. Histone serotonylation in dorsal raphe nucleus contributes to stress- and antidepressant-mediated gene expression and behavior. Nat Commun. 2024;15(1). 10.1038/s41467-024-49336-4. 10.1038/s41467-024-49336-4 PMC11176395 38871707

[73] Müller N, Schwarz MJ. The immune-mediated alteration of serotonin and glutamate: towards an integrated view of depression. Mol Psychiatry. 2007;12(11):988–1000. 10.1038/sj.mp.4002006. 17457312 10.1038/sj.mp.4002006

[74] Miller AH. Conceptual confluence: the kynurenine pathway as a common target for ketamine and the convergence of the inflammation and glutamate hypotheses of depression. Neuropsychopharmacology. 2013;38(9):1607–8. 10.1038/npp.2013.140. 23857540 10.1038/npp.2013.140 PMC3717552

[75] Wang B, Lian YJ, Su WJ, Peng W, Dong X, Liu LL, et al. HMGB1 mediates depressive behavior induced by chronic stress through activating the kynurenine pathway. Brain Behav Immun. 2018;72:51–60. 29195782 10.1016/j.bbi.2017.11.017

[76] Laumet G, Zhou W, Dantzer R, Edralin JD, Huo XJ, Budac DP, et al. Upregulation of neuronal kynurenine 3-monooxygenase mediates depression-like behavior in a mouse model of neuropathic pain. Brain Behav Immun. 2017;66:94–102. 28709913 10.1016/j.bbi.2017.07.008 PMC5650931

[77] Mithaiwala MN, Santana-Coelho D, Porter GA, O’connor JC. Neuroinflammation and the Kynurenine Pathway in CNS Disease: Molecular Mechanisms and Therapeutic Implications. Cells. 2021;10(6). 10.3390/cells10061548. 10.3390/cells10061548 PMC8235708 34205235

[78] Réus GZ, Jansen K, Titus S, Carvalho AF, Gabbay V, Quevedo J. Kynurenine pathway dysfunction in the pathophysiology and treatment of depression: Evidences from animal and human studies. J Psychiatr Res. 2015;68:316–28. 10.1016/j.jpsychires.2015.05.007. 26028548 10.1016/j.jpsychires.2015.05.007 PMC4955923

[79] Savitz J, Dantzer R, Meier TB, Wurfel BE, Victor TA, McIntosh SA, et al. Activation of the kynurenine pathway is associated with striatal volume in major depressive disorder. Psychoneuroendocrinology. 2015;62:54–8. 26232650 10.1016/j.psyneuen.2015.07.609 PMC4637239

[80] Sublette ME, Galfalvy HC, Fuchs D, Lapidus M, Grunebaum MF, Oquendo MA, et al. Plasma kynurenine levels are elevated in suicide attempters with major depressive disorder. Brain Behav Immun. 2011;25(6):1272–8. 10.1016/j.bbi.2011.05.002. 21605657 10.1016/j.bbi.2011.05.002 PMC3468945

[81] Fuertig R, Azzinnari D, Bergamini G, Cathomas F, Sigrist H, Seifritz E, et al. Mouse chronic social stress increases blood and brain kynurenine pathway activity and fear behaviour: Both effects are reversed by inhibition of indoleamine 2,3-dioxygenase. Brain Behav Immun. 2016;54:59–72. 26724575 10.1016/j.bbi.2015.12.020

[82] Sato M, Okuno A, Suzuki K, Ohsawa N, Inoue E, Miyaguchi Y, et al. Dietary intake of the citrus flavonoid hesperidin affects stress-resilience and brain kynurenine levels in a subchronic and mild social defeat stress model in mice. Biosci Biotechnol Biochem. 2019;83(9):1756–65. 31119994 10.1080/09168451.2019.1621152

[83] Câmara AB, Brandão IA. The interplay between Kynurenine and Nociceptin/Orphanin FQ pathways can be related to depressive-like phenotype. Eur J Pharmacol. 2025;1001:177766. 10.1016/j.ejphar.2025.177766. 40412744 10.1016/j.ejphar.2025.177766

[84] Gracia-Rubio I, Moscoso-Castro M, Pozo OJ, Marcos J, Nadal R, Valverde O. Maternal separation induces neuroinflammation and long-lasting emotional alterations in mice. Prog Neuropsychopharmacol Biol Psychiatry. 2016;65:104–17. 26382758 10.1016/j.pnpbp.2015.09.003

[85] Nikkheslat N, Zajkowska Z, Legido-Quigley C, Xu J, Manfro PH, Souza L, et al. Sex-Specific Alterations of the Kynurenine Pathway in Association With Risk for and Remission of Depression in Adolescence. Biol Psychiatry. 2025;98(7):549–57. 40131256 10.1016/j.biopsych.2024.11.020

[86] Eskelund A, Budac DP, Sanchez C, Elfving B, Wegener G. Female flinders sensitive line rats show estrous cycle-independent depression-like behavior and altered tryptophan metabolism. Neurosci 2016 Aug 4:329337–48. 10.1016/j.neuroscience.2016.05.024 10.1016/j.neuroscience.2016.05.024 27210075

[87] Morgese MG, Schiavone S, Maffione AB, Tucci P, Trabace L. Depressive-like phenotype evoked by lifelong nutritional omega-3 deficiency in female rats: crosstalk among kynurenine, toll-like receptors and amyloid beta oligomers. Brain Behav Immun 2020 Jul:87:444–54. 10.1016/j.bbi.2020.01.015. 10.1016/j.bbi.2020.01.015 31987923

[88] Li CC, Ye F, Xu CX, Jiang N, Chang Q, Liu XM, Pan RL. Tryptophan-kynurenine metabolic characterization in the gut and brain of depressive-like rats induced by chronic restraint stress. J Affect Disord 2023 May 1:328:273–86. 10.1016/j.jad.2023.02.008. 10.1016/j.jad.2023.02.008 36746244

[89] Laugeray A, Launay JM, Callebert J, Surget A, Belzung C, Barone PR. Evidence for a key role of the peripheral kynurenine pathway in the modulation of anxiety- and depression-like behaviours in mice: focus on individual differences. Pharmacol Biochem Behav. 2011;98(1):161–8. 10.1016/j.pbb.2010.12.008. 21167857 10.1016/j.pbb.2010.12.008

[90] Takahashi A, Chung JR, Zhang S, Zhang H, Grossman Y, Aleyasin H, Flanigan ME, Pfau ML, Menard C, Dumitriu D, Hodes GE, McEwen BS, Nestler EJ, Han MH, Russo SJ. Establishment of a repeated social defeat stress model in female mice. Sci Rep. 2017;7(1):12838. 10.1038/s41598-017-12811-8. 28993631 10.1038/s41598-017-12811-8 PMC5634448

[91] Yohn CN, Dieterich A, Bazer AS, Maita I, Giedraitis M, Samuels BA. Chronic Non-Discriminatory Social Defeat Is an Effective Chronic Stress Paradigm for Both Male and Female Mice. Neuropsychopharmacology. 2019;44(13):2220–9. 10.1038/s41386-019-0520-7. 31493767 10.1038/s41386-019-0520-7 PMC6898575

[92] Smith A, Hyland L, Al-Ansari H, Watts B, Silver Z, Wang L, Dahir M, Akgun A, Telfer A, Abizaid A. Metabolic, neuroendocrine and behavioral effects of social defeat in male and female mice using the chronic non-discriminatory social defeat stress model. Horm Behav. 2023;155:105412. 10.1016/j.yhbeh.2023.105412. 37633226 10.1016/j.yhbeh.2023.105412

[93] Yin W, Gallagher NR, Sawicki CM, McKim DB, Godbout JP, Sheridan JF. Repeated social defeat in female mice induces anxiety-like behavior associated with enhanced myelopoiesis and increased monocyte accumulation in the brain. Brain Behav Immun 2019 May:78:131–42. 10.1016/j.bbi.2019.01.015. 10.1016/j.bbi.2019.01.015 PMC6488440 30684650

[94] Prakash N, Stark CJ, Keisler MN, Luo L, Der-Avakian A, Dulcis D. Serotonergic Plasticity in the Dorsal Raphe Nucleus Characterizes Susceptibility and Resilience to Anhedonia. J Neurosci. 2020;40(3):569–84. 10.1523/JNEUROSCI.1802-19. 31792153 10.1523/JNEUROSCI.1802-19.2019 PMC6961996

